# Pressure of Invasive Alien Species *Trachemys scripta* on Native Species Under Future Climate Change Scenarios

**DOI:** 10.1002/ece3.73084

**Published:** 2026-02-11

**Authors:** Nilgün Kaya, Harun İnci, İrem Şarlak, Tuğçe Yetim, Ceren Nur Özgül, Oya Özuluğ, Murat Tosunoğlu

**Affiliations:** ^1^ Department of Biology, Faculty of Science Çanakkale Onsekiz Mart University Çanakkale Turkiye; ^2^ Department of Biology, Faculty of Science Istanbul University İstanbul Turkiye; ^3^ Department of Biology, Institute of Science Istanbul University İstanbul Turkiye

**Keywords:** conservation, habitat competition, invasive, *Trachemys scripta*, Türkiye

## Abstract

Throughout the world, climate change is having many adverse impacts, ranging from the decline of biodiversity to the economic downturn. Increasing temperature will continue to affect microorganisms and ecosystems in a very wide range. In order to mitigate the severity of this irreversible process, it would be helpful to analyze the anticipated scenarios for the coming years. For this purpose, the invasive alien species *Trachemys scripta* and the native species *Emys orbicularis* , *Mauremys caspica* and *Mauremys rivulata* in Türkiye were projected with five different climate models (ACCESS‐CM2, BCC‐CSM2‐MR, CNRM‐ESM2‐1, GISS‐E2‐1‐G, and MIROC6) for the years 2050, 2070, and 2090. Suitable habitat areas, habitat expansions, and habitat contractions of species with climate change were modeled. Based on the results of these models, it appears that habitat expansions in the future will probably result in an increase in competition between native and invasive species. Due to habitat contraction in the west, the *T. scripta* species is expected to migrate toward the coast, which may lead to population declines for *E. orbicularis* and *M. rivulata* , especially along the Mediterranean coast. Furthermore, *M. caspica* , which is distributed in the east, is likely to move toward the western and southern regions due to climate change, where it could compete for habitat with *T. scripta* as it experiences habitat contraction in the north. This suggests that climate change and the impact of invasive species will lead to habitat loss for native species in the future. Considering this data, it is recommended to increase collection and monitoring efforts in coastal areas where the *T. scripta* species is currently densely distributed in order to mitigate the occurrence of this predicted scenario in the future.

## Graphical abstract

In this study, ecological niche models are provided for *Trachemys scripta* and its competing species in Turkey. In 20‐year intervals, the map indicates the future habitat areas suitable for the invasive species as well as its competitors. The results of this study have led to a determination of the regions where invasive species are likely to be distributed in the coming years as a result of climate change.

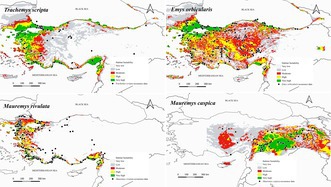

## 1 Introduction

The term “invasive alien species” refers to species that have been translocated by humans into environments outside their natural range, in which they settle and distribute, negatively affecting the dynamics of local ecosystems (Chinchio et al. 2020). Many negative effects (economic and ecological) are caused by invasive species. The irreversible loss of species is one of the major consequences of biological invasions (Mollot et al. 2017).

The reptile trade has historically been dominated by a relatively small number of common and popular reptiles, despite involving thousands of species (Marshall et al. 2020; Valdez 2021). Online trade of live animals, including the *Trachemys scripta* (Thunberg, 1792) (red‐eared slider turtle), has contributed to the spread of invasive species (Kikillus et al. 2012). Over the last century, red‐eared sliders, *T. scripta elegans* , were one of the most traded reptiles in the world (Easter and Carter 2024; Pérez‐Santigosa et al. 2011). In 2005, an estimated $24 billion was traded worldwide in legal wildlife trade, excluding fisheries and timber (Robinson et al. 2015). The trade of live reptiles into the EU alone in 2010 totaled 4.3 million USD, accounting for 22% of all live imports in value, exceeding the trade of mammals (Robinson et al. 2015). Unfortunately, quantifying the full extent of the reptile trade is challenging as domestic and unregulated trade is much harder to track and document (Janssen and Shepherd 2018; Marshall et al. 2020). As a result of this high commercial return, wildlife trade is on the increase. By monitoring online sales of exotic species, we can gain valuable demographic information and determine propagule pressure through the pet‐release route (Kikillus et al. 2012).

Invasions generally rely on two characteristics to be successful. The first is characterized by traits that allow non‐native species to colonize new habitats. A second set of characteristics relates to the ability of a colonized species to adapt to its new aquatic environment (Ehrlich 1986). Species with large eggs or parental care in aquatic habitats have been found to be more successful invaders. Furthermore, the rate of spread to nearby areas with similar habitats also indicates the long‐term success of invasive species (Hanski 1999). The success of the species' establishment, rather than its rate of spread, is crucial for the initial stages of invasion (Kolar and Lodge 2000). The invasion also leads to competition with native species when it is successful. As introduced sliders and native terrapins overlap in diet and feeding areas, devote a high percentage of their time budget to basking, and coincide with the breeding season, competition for food, basking sites, or nesting sites is likely to occur (Cadi and Joly 2004). Compared with native turtles, *T. scripta* may possess a larger adult body size, a more varied diet, a higher fertility, and a greater tolerance for pollution and human activity (Gibbons 1990).

It is also important to note that invasive species may carry pathogens such as viruses and bacteria that can cause disease and even death (Telfer and Bown 2012). There are three possible locations from which invasive species may harbor parasites: (i) the native range of the invasive species, (ii) a location intermediate from its native range to its current location, and (iii) the area where the invasive species becomes established (Chalkowski et al. 2018). Pathogens such as these can lead to serious population losses in native species, thus resulting in biodiversity loss and ecosystem degradation (Chinchio et al. 2020). As a result of climate change and increased commercial mobility, these pathogens are even easier to transport.

Apart from invasive species, climate change is the most pressing issue of our time. From biological systems to the economy, climate change is expected to have a wide range of impacts. A change in climate may affect invasive species in three ways: (i) altering climates directly affect individuals; (ii) altering resource availability and interaction with other species indirectly; and (iii) other factors, such as human influences, which may alter the environment for invasive species as well (Finch et al. 2021). In combination, each of these drivers of change compounded the negative impacts of the prior, and the interaction between the two threats presents an even greater challenge for conservationists on the ground and policy makers alike (Mainka and Howard 2010). As a result, the social and economic impacts of climate change and invasive species, which are already substantial, will become even more pronounced (Mainka and Howard 2010).

Türkiye is a country with different continental formations within the Palearctic region, with a climatic and vegetation structure that varies from west to east, and with various barriers and three hotspots (the Mediterranean, the Iran‐Anatolian, and the Caucasus) (Bilgin 2011). At the same time, due to its zoogeographic feature, terrestrial and marine connections that enable the passage of species from Europe and Asia facilitate the passage of invasive species into the country. The extensive damming in Türkiye, as in other regions of the world, has degraded water quality, prevented native species from moving, and facilitated the invasion of foreign species (Şekercioğlu et al. 2011). Additionally, Türkiye's trade through both imports and export contributes to its vulnerability to biological invasions (Aksu et al. 2021; Emiroğlu et al. 2020). Economic benefits and ease of cultivation have been influential factors in the intentional introduction of invasive species into the country (Tarkan et al. 2020). Given the country's diverse ecosystems and dynamic economy, economic impact assessments are also being conducted to address the increasing number of non‐native invasive species (Tarkan et al. 2024). Simultaneously, efforts are underway in Türkiye to identify and control invasive species (Çinar et al. 2021; Karaer et al. 2015).

In this study, we aimed to reveal the changes in the suitable habitat areas between 2050 and 2090 under climate change using data from the invasive exotic *T. scripta* species and the native species *Emys orbicularis* (Linnaeus, 1758), *Mauremys caspica* (Gmelin, 1774) and *Mauremys rivulata* (Valenciennes, 1833) in Türkiye. Hence, the habitat expansions and contractions of the species were calculated and refined, and the possible encounters of the invasive species with the local species and the possible habitat competition areas were determined. Furthermore, the aim is to provide data for species action plans by identifying the potential habitat expansion areas of the *T. scripta* species.

## 2 Materials and Methods

### 2.1 Occurrence Data

A total of 1037 presence data were collected for the species of *T. scripta* , *M. caspica* , *M. rivulata* , and *E. orbicularis* from various sources. Presence data (179 records) for *T. scripta* were obtained from field surveys (7 record), literatures (10 records) (Çiçek and Ayaz 2015; Eldeleklioğlu et al. 2023; Koç‐Gür et al. 2021; Şahin 2021; Uysal et al. 2018; Yakin et al. 2024), and various databases (162 records) (Turkish Frog and Reptile Observation and Photography Society (Available: http:www.turkherptil.org [Accessed April 25, 2024]), GBIF: The Global Biodiversity Information Facility (2023) GBIF Occurrence Download Website Available: https://doi.org/10.15468/dl.66m4jd [Accessed December 9, 2023]). 20 Records for *M. caspica* were obtained from field surveys (1 record), literatures (19 records) (Adızel et al. 2017; Cihan et al. 2003; Göçmen et al. 2008; Özcan and Sarıeyyüpoğlu 2009; Özuslu and Tel 2010; Yıldız et al. 2018; Yalçınkaya et al. 2022; Yıldız et al. 2023), and various databases (112 records) (Turkish Frog and Reptile Observation and Photography Society (Available: http:www.turkherptil.org [Accessed April 20, 2024]), GBIF: The Global Biodiversity Information Facility (2024) GBIF Occurrence Download Website Available: https://doi.org/10.15468/dl.5wu6kj [Accessed July 9, 2024]). 457 Records for *M. rivulata* were obtained from collection of museums (5 records) (Zoological Museum, University of Çanakkale Onsekiz Mart Biology Department), field surveys (65 records), literatures (17 records) (Ayaz and Budak 2008; Bayrakcı et al. 2016; Çakmak et al. 2017; Keleş 2019; Özcan and Üzüm 2014; Özgül et al. 2022; Yakin et al. 2024), and various databases (370 records) (Turkish Frog and Reptile Observation and Photography Society (Available: http:www.turkherptil.org [Accessed: April 16, 2024]), GBIF: The Global Biodiversity Information Facility (2024) GBIF Occurrence Download Website Available: https://doi.org/10.15468/dl.avy8dy [Accessed: July 9, 2024]). 269 Records for *E. orbicularis* were obtained from collection of museum (3 records) (Zoological Museum, University of Istanbul, Biology Department), field surveys (39 records), literatures (92 records) (Akman et al. 2020; Ayaz and Budak 2007; Ayaz et al. 2008; Ayaz et al. 2017; Bayrakcı and Ayaz 2014; Bayrakcı et al. 2016; Bayrakcı et al. 2021; Çakmak et al. 2017; Çiçek and Ayaz 2011; Fritz et al. 2009; Sarikaya et al. 2017; Soylu et al. 2006; Şahin 2021; Yıldız et al. 2019), and database (135 records) (GBIF: The Global Biodiversity Information Facility (2024) GBIF Occurrence Download Website Available: https://doi.org/10.15468/dl.fstcny [Accessed: November 12, 2024]) (Appendix S1, Table S1). The record dates of presence data ranged from the 1980s to 2024.

### 2.2 Ecological Niche Modeling

In order to model ecological niches, the software Maxent v. 3.4.1 was used (Phillips et al. 2017), as it enables presence information to be used (not absence information or non‐detection information). The variables and model settings were selected in Wallace (v. 1.1.3) software, a GUI application that accesses R‐scripted modern workflows (Kass et al. 2018). As part of the spatial filtering process, multiple presence records within a 1‐km distances were merged into one record. Bioclimatic data were downloaded from WorldClim version 2.1 database with a spatial resolution of 2.5 arc‐min for present (1970–2000), and future (2050, 2070, and 2090) with SSP1‐2.6 (the minimum scenario for GHG emissions) and SSP5‐8.5 (the highest scenario for GHG emissions). Thirteen (Bio1, Bio2, Bio3, Bio4, Bio7, Bio9, Bio10, Bio11, Bio12, Bio14, Bio15, Bio17, and Bio18) of the 19 bioclimatic variables and topographic variables (elevation, slope, terrain roughness index) that were thought to affect the distribution of the species were used. The topographic data were downloaded from the EarthEnv project (Amatulli et al. 2018). For selecting the background extent, a 2 degree bounding box was applied. 100,000 of background points were sampled. The presence data were partitioned using spatial partitioning (Block, *k* = 4) in which records are divided into four equal bins based on latitudinal and longitudinal lines. We tested L (linear), LQ (linear and quadratic), H (hinge), LQH (linear, quadratic, and hinge), and LQHP (linear, quadratic, hinge, and product) models for each candidate model with five values of regularization multiplier (1 to 10 in increments of 1). A candidate model was tested with five models; four of these models were developed iteratively. As a result, three out of four bins were used for model training, and the withheld bin was used for model testing in order to determine threshold‐independent (AUCTEST and AUCDIFF) and threshold‐dependent models (OR10 (10% training omission rate)). In addition, one model calculated the Akaike information criterion corrected for small sample sizes (AICc) based on the entire dataset. By accounting for both model fit and complexity, AICc improves the Akaike information criterion (AIC) in order to find a model that fits the data well without being overly complex (Li et al. 2025). Among the candidate models, the model was selected with an AUC valued higher than 0.80. Furthermore, lower delta.AICc (Delta Akaike information criterion corrected) scores indicate improved models under the AICc criterion (Ogasawara 2016). The final model was projected onto present and future conditions of the sampling area. The future climate scenarios for habitat suitability in the 2050s, 2070s, and 2090s we used Coupled Model Intercomparison Projects (CMIP6). Five scenarios (ACCESS‐CM2, BCC‐CSM2‐MR, CNRM‐ESM2‐1, GISS‐E2‐1‐G, MIROC6) of CMIP6 were performed. We mapped each scenario by the QGIS version 3.38.2 map program. Classifications ranged from 0 (low suitability) to 1 (high suitability) in bioclimatic suitability maps.

To evaluate the model's performance and significance, ROC analysis was performed (Peterson et al. 2008) using the software ntbox (NicheToolBox) version v1.0.0 (Osorio‐Olvera et al. 2020). This analysis was conducted using the following settings: proportion of omission = 0.05, percentage of random points = 50, and number of bootstrap iterations = 10,000.

In order to calculate range expansion, suitable areas in both, total suitable areas, contraction, and unsuitable areas in both, SDMtoolbox version 2.6 (Brown et al. 2017) was used. A buffer zone of 200 km has been created for each of the species' maps. High and very high suitability were defined as suitable bioclimatic areas according to the “10 Percentile training presence” threshold. ArcGIS 10.8 software was used for GIS operations.

## 3 Results

The final model was constructed using the input data for each species separately, including Bio1, Bio2, Bio3, Bio4, Bio7, Bio9, Bio10, Bio11, Bio12, Bio14, Bio15, Bio17, Bio18, elevation, slope, and terrain roughness index. Models used all of 16 input variables and performed better than a random prediction for species (statistics for AUC (Area Under the Curve) ratio, model for *T. scripta* : mean ± SD = 1.24 ± 0.03, range = 1.14–1.41, *p* < 0.001, model for *E. orbicularis* : mean ± SD = 1.36 ± 0.03, range = 1.29–1.45, *p* < 0.001, model for *M. caspica* : mean ± SD = 1.5 ± 0.1, range = 1.3–1.72, *p* < 0.001, model for *M. rivulata* : mean ± SD = 1.59 ± 0.06, range = 1.38–1.77, *p* < 0.001). The performance of the final model was chosen based on the value of the area under the curve (AUC) greater than 0.8 (Table 1).

**TABLE 1:** Analyzing the performance of candidate models by their AUC, OR10, and deltaAICc values (rm: regularization multiplier, fc: feature classes (L = Linear, Q = Quadratic, H = Hinge, P = Product), auc.train: AUC calculated using all occurrence localities, auc.val.avg.: mean deviation of the *k* validation AUCs, or.10p.avg. and or.10p.sd: mean and standard deviation of all test 10pct omission rates, delta AICc: the difference between the lowest AICc and each AICc).

Species	rm	fc	auc.train	auc.val.avg	or.10p.avg	or.10p.sd	delta.AICc
*Trachemys scripta*	5	LQHP	0.867	0.833	0.175	0.236	0
*Emys orbicularis*	5	LQHP	0.843	0.806	0.2067	0.253	0
*Mauremys caspica*	4	LQHP	0.885	0.8257	0.2018	0.0972	5.661
*Mauremys rivulata*	4	LQHP	0.924	0.914	0.116	0.198	0

**FIGURE 1:**
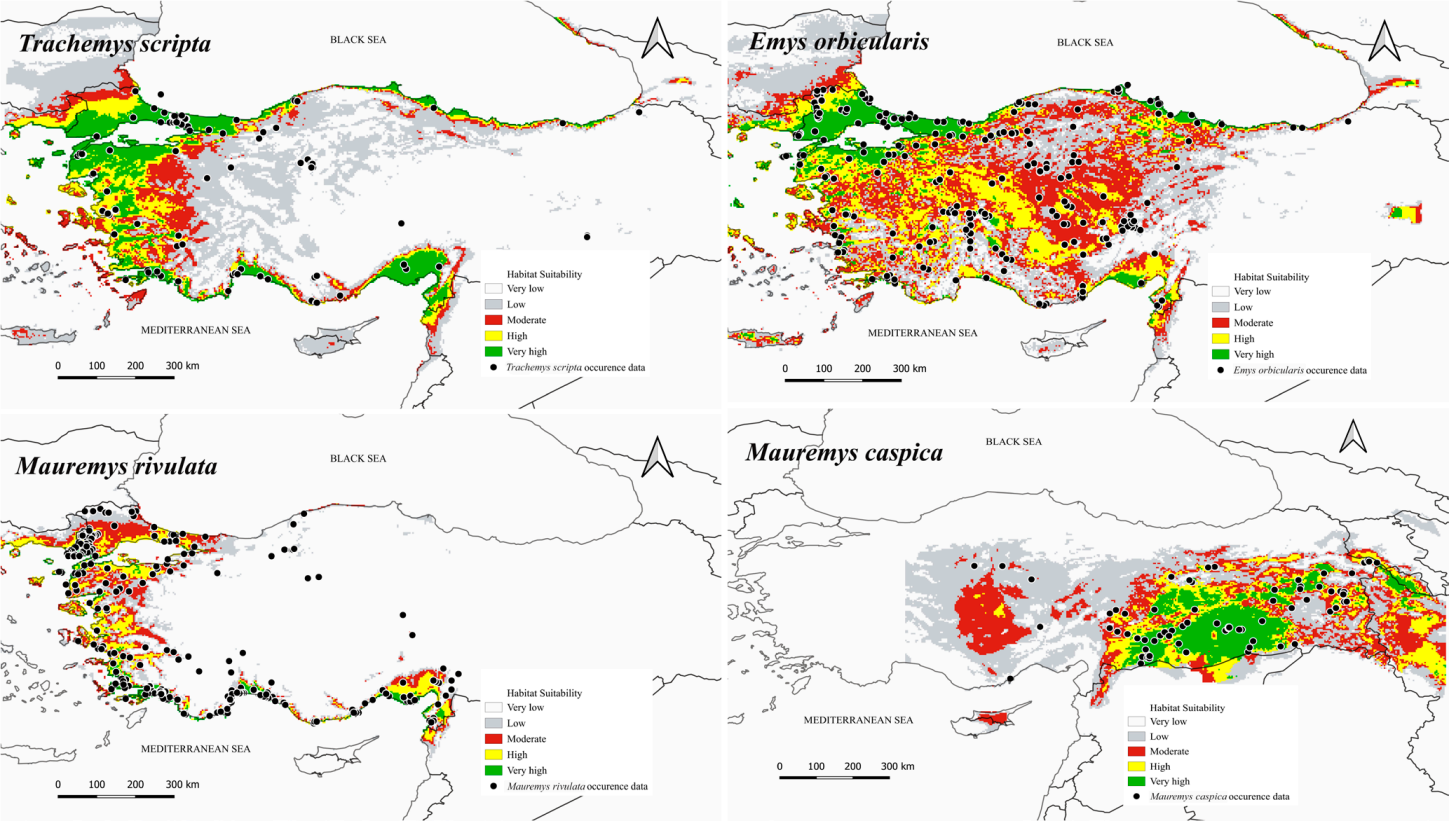
The habitat suitabilities of *Trachemys scripta* , *Emys orbicularis* , *Mauremys caspica* , and *Mauremys rivulata* under present conditions (1970–2000).

**FIGURE 2:**
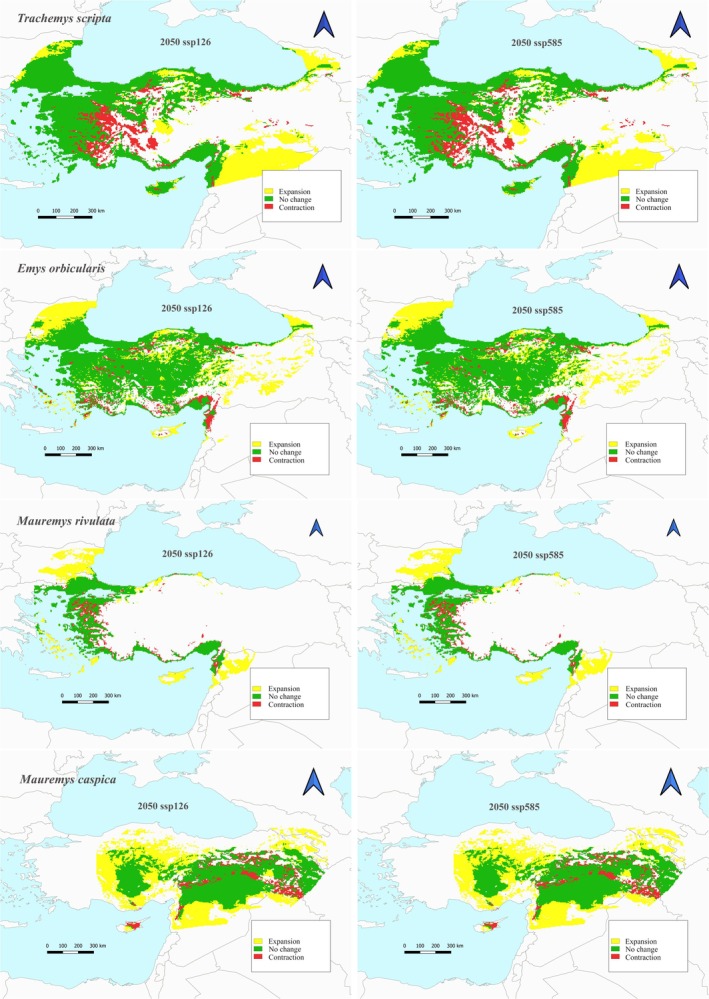
Habitat suitability under future (2050) conditions for each scenario (ACCESS‐CM2, BCC‐CSM2‐MR, CNRM‐ESM2‐1, GISS‐E2‐1‐G, MIROC6 with SSP1‐2.6 (the minimum scenario for GHG emissions) and SSP5‐8.5 (the highest scenario for GHG emissions)) for *Trachemys scripta* , *Emys orbicularis* , *Mauremys caspica* , and *Mauremys rivulata* .

**FIGURE 3:**
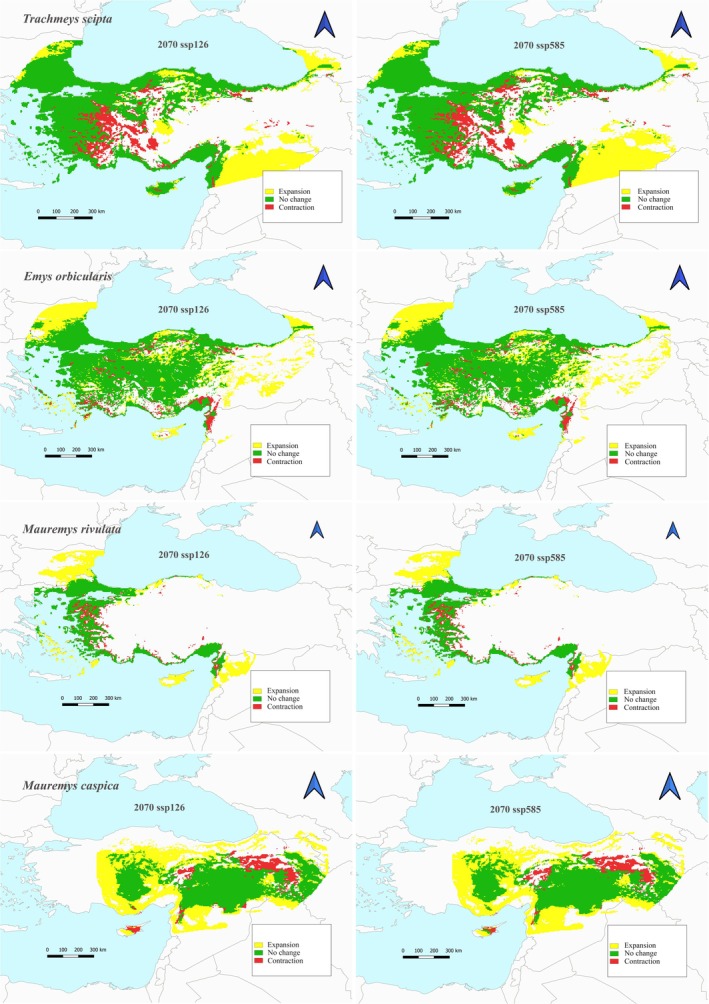
Habitat suitability under future (2070) conditions for each scenario (ACCESS‐CM2, BCC‐CSM2‐MR, CNRM‐ESM2‐1, GISS‐E2‐1‐G, MIROC6 with SSP1‐2.6 (the minimum scenario for GHG emissions) and SSP5‐8.5 (the highest scenario for GHG emissions)) for *Trachemys scripta* , *Emys orbicularis, Mauremys caspica*, and *Mauremys rivulata* .

**FIGURE 4:**
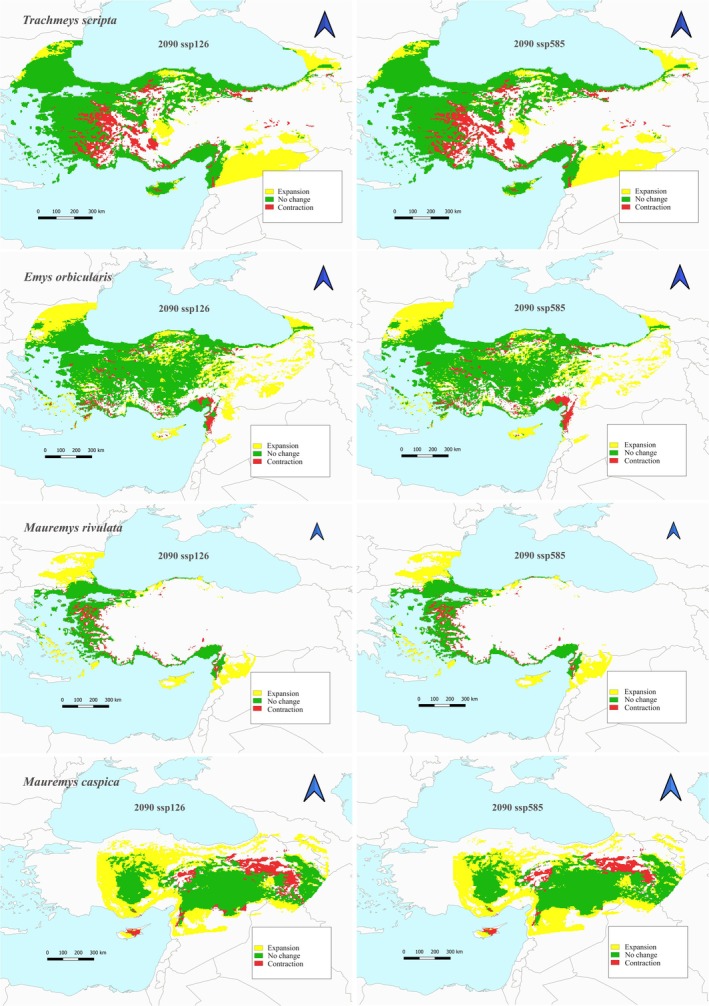
Habitat suitability under future (2090) conditions for each scenario (ACCESS‐CM2, BCC‐CSM2‐MR, CNRM‐ESM2‐1, GISS‐E2‐1‐G, MIROC6 with SSP1‐2.6 (the minimum scenario for GHG emissions) and SSP5‐8.5 (the highest scenario for GHG emissions)) for *Trachemys scripta* , *Emys orbicularis* , *Mauremys caspica* , and *Mauremys rivulata* .

The current suitable habitat areas and localities of the species are shown in Figure 1. The total suitable habitat area of *T. scripta* is currently 399,256 km^2^. In future climate scenarios with SSP1‐2.6 and SSP5‐8.5, the species is estimated to have an average habitat contraction of around 16% in 2050, 2070, and 2090 (Figures 2, 3, 4, Table 2). The habitat expansion is around 34% on average. However, only in the 2090 SSP5‐8.5 scenario, this expansion is estimated to be around 31.98%.

**TABLE 2:** Suitable bioclimatic areas (km^2^) that are suitable for *Trachemys scripta, Emys orbicularis, Mauremys caspica*, and *Mauremys rivulata* under present (1970–2000), and future scenarios (for each scenario, 2050, 2070, and 2090 with SSP1‐2.6 and SSP5‐8.5).

		Present	2050		2070		2090	
			SSP1‐2.6	SSP5‐8.5	SSP1‐2.6	SSP5‐8.5	SSP1‐2.6	SSP5‐8.5
*Trachemys scripta*	Expansion		138,559	136,704	138,886	136,327	136,941	127,697
	Suitable areas in both	399,256	332,843	334,968	333,997	333,733	332,769	333,443
	Total suitable areas		471,402	471,672	472,883	470,060	469,710	461,140
	Contraction		66,414	64,289	65,259	65,523	66,487	65,813
	Unsuitable areas in both	794,276	655,717	657,572	655,390	657,949	657,335	666,579
*Emys orbicularis*	Expansion		162,841	163,581	169,020	161,395	168,957	153,622
	Suitable areas in both	421,469	388,276	388,599	388,829	388,072	388,692	384,426
	Total suitable areas		551,117	552,180	557,849	549,467	557,649	538,048
	Contraction		33,192	32,869	32,639	33,396	32,776	37,042
	Unsuitable areas in both	748,065	585,223	584,483	579,044	586,670	579,107	594,442
*Mauremys caspica*	Expansion		225,957	222,839	233,069	216,744	230,129	223,126
	Suitable areas in both	296,242	247,284	248,553	247,277	251,149	245,981	250,217
	Total suitable areas		473,241	471,392	480,346	467,893	476,110	473,343
	Contraction		48,958	47,689	48,964	45,093	50,260	46,025
	Unsuitable areas in both	705,987	480,029	483,148	472,918	489,243	475,858	482,861
*Mauremys rivulata*	Expansion		81,659	85,923	86,000	88,329	85,713	92,090
	Suitable areas in both	146,192	122,840	124,802	124,752	125,283	124,485	126,027
	Total suitable areas		204,499	210,726	210,753	213,612	210,199	218,117
	Contraction		23,352	21,390	21,440	20,909	21,707	20,165
	Unsuitable areas in both	661,353	579,694	575,429	575,353	573,023	575,640	569,263

The total suitable habitat area of *E. orbicularis* species is currently 421,469 km^2^. In future climate scenarios according to SSP1‐2.6 and SSP5‐8.5, the species is estimated to have a habitat contraction of approximately 7.7% in 2050, 2070, and 2090 (Figures 2, 3, 4, Table 2). However, only in the 2090 SSP5‐8.5 scenario, this contraction is estimated to be approximately 8.79%. The maximum habitat expansion is estimated to be 40.1% in the 2070 with SSP1‐2.6 scenario. The lowest habitat expansion is predicted to be 36.45% in 2090 with SSP5‐8.5 scenario.

The total suitable habitat area of *M. caspica* species is currently 296,242 km^2^. According to the 2070 SSP5‐8.5 scenario, the lowest habitat contraction is calculated as 15.22% (Figure 3, Table 2). Habitat expansion is predicted to be the lowest 73.16% according to the 2070 SSP5‐8.5 scenario and the highest 78.68% in the 2070 SSP1‐2.6 scenario. In future climate scenarios according to the 2090 SSP1‐2.6 scenario, habitat contraction is the highest 16.97% (Figure 4, Table 2).

The total suitable habitat area of *M. rivulata* is currently estimated to be 146,192 km^2^. In future climate scenarios, habitat contraction is expected to be the lowest with an estimated 13.79% according to the 2090 SSP5‐8.5 scenario, while it is expected to be the highest with an estimated 15.97% according to the 2050 SSP1‐2.6 scenario (Figures 2 and 4, Table 2). Habitat expansion is estimated to be the lowest with an estimated 55.86% with the 2050 SSP1‐2.6 scenario. On the other hand, the scenario in which habitat expansion will be the highest is the 2090 SSP5‐8.5 scenario with an increase of 62.99%.

## 4 Discussion

Since the 1950s, *T. scripta* , which is native to the eastern US and northeast Mexico, has been traded throughout the world (Ficetola et al. 2012). Its low price and ease of care quickly made it one of the most popular pets in the pet trade (Ficetola et al. 2012). They have rapidly spread, especially in temperate climates, due to people releasing unwanted pets into nearby parks, gardens, streams, and ponds (Semenov 2010). Physical barriers in terrestrial ecosystems often restrict species movement. In contrast, species movement is faster in freshwater ecosystems. This is likely due to the connections between aquatic systems through rivers and canals, which allow species to disperse more quickly and efficiently than on land (Leuven et al. 2009). *Trachemys scripta* is a rapidly spreading species in Türkiye. Alongside this, biological control studies for this species have also begun. To implement an effective biosecurity policy, it is essential to determine future potential species distributions (Hodson et al. 2025). Therefore, this study is to provide data on the potential future distribution areas of the invasive species and native turtle species it competes with in Türkiye, in order to develop effective solutions.

The invasive alien *T. scripta* species is distributed in regions of Türkiye other than the central and eastern parts. With climate change, it is predicted that habitat contraction will occur in the western regions by 2050, while habitat expansion will occur eastward along the coast. Model results show that habitat contraction in the west will be concentrated in the Lake District and its surroundings. The Lake Region is composed of nine large and more than 20 small lakes, covering an area of approximately 40,000 km^2^ (Kazancı and Roberts 2019). Data shows that the region has experienced a decrease in rainfall and an increase in drought in recent years (Soydan Oksal and Beden 2024). Drought is a serious problem and may have negative impacts on biodiversity, as it affects agricultural activities and causes habitat degradation. Drought scenarios for Türkiye indicate that many wetlands and their surrounding areas will experience drought in the coming years (Güney 2024). Climate change scenarios also suggest that the Lake Region will face long‐term and moderate droughts in the future (Çolak et al. 2022). It is estimated that the habitat loss for the *T. scripta* species will be over 64,000 km^2^ in 2050, according to the SSP1‐2.6 and SSP5‐8.5 scenarios. In contrast, the habitat loss for the *E. orbicularis* species is expected to be between 33,192 and 32,869 km^2^, with this loss concentrated in the eastern Mediterranean. However, despite the habitat loss, expansion in suitable habitat areas for this species is expected to be concentrated in the eastern region where the species is not currently distributed. Habitat expansion constitutes 38.6% of the current suitable habitat area. This indicates a significant increase in suitable habitat. If the invasive *T. scripta* species continues to exert habitat pressure on *E. orbicularis* , with climate change, the species' distribution is expected to shift eastward. The *M. rivulata* species has a more restricted distribution compared with the other species. The species is mainly distributed along the coastal areas of the Marmara, Aegean, and Mediterranean Seas. According to 2050 scenarios, while suitable habitat areas are expected to contract in the west, there is a prediction of habitat expansion towards the north and along the Black Sea coast. In addition to the contraction of suitable habitats, the dominance of the *T. scripta* species in these areas is expected to negatively affect the distribution and population of this species. The *M. caspica* species is distributed in the eastern part of Türkiye and Central Anatolia. According to 2050 scenarios, while the habitat in the east is expected to contract, the habitat is predicted to expand westward and southward. If the species moves as predicted by the scenarios, it will inevitably face habitat competition with the invasive species in these regions. The fact that habitat expansion occurs in the eastern Mediterranean for both species suggests that the distribution of the *M. caspica* species could be defined in this area.

The *T. scripta* species exhibits similarities in the 2070 with SSP1‐2.6 scenario compared with the 2050 scenarios. However, with the SSP5‐8.5 scenario, the species is observed to increase its northward habitat expansion in the east. It is predicted that the *M. caspica* species, which is distributed in this region, will experience habitat contraction in the northern regions in the 2070 scenarios. In such a scenario, it is expected that the populations of this species will migrate towards the southern regions with more suitable habitats. If this scenario materializes, habitat competition with the invasive species becomes inevitable. It is known that in freshwater environments, the native species and the invasive *T. scripta* species do not differ significantly in terms of food preferences (Pérez‐Santigosa et al. 2011). However, it is known that the invasive *T. scripta* species has a much broader food preference (Pérez‐Santigosa et al. 2011) and dominates over native species in food competition (Cadi and Joly 2004). Additionally, it has been observed that these cold‐blooded creatures, when sharing the same habitat, cause the native species to reduce their sunbathing hours due to the *T. scripta* species' longer basking periods (Polo‐Cavia et al. 2010). The 2070 scenarios for *Emys orbicularis* and *M. rivulata* species show similarities to the 2050 scenarios.

In the 2090 scenarios, both scenarios show a habitat contraction towards the coastal regions of Western Anatolia for the *T. scripta* species. This indicates that the species will either move to the northern or southern coastal regions. The *E. orbicularis* species, on the other hand, is expanding its habitat northward and eastward according to the 2090 scenarios. It is sharing suitable habitat areas in the coastal regions with the invasive species. The species with the lowest distribution among the native species is *M. rivulata* . According to the 2090 scenarios, it is predicted that the habitat area of this species will be limited to the coastal area. In this area, the dominance of the *T. scripta* species is also observed. Accordingly, if the predicted scenario occurs, it is estimated that there will be significant habitat contractions in the distribution of the species along the coastline. The greatest habitat loss likely occurs in the Aegean region in the west. In the 2090 scenarios, it is observed that the habitat contraction of the *M. caspica* species will be extensive in the north and east. In such a scenario, the species is expected to move westward and southward. Modeling results show that there will be habitat expansion in the west and south. As a result of these scenarios, *E. orbicularis* and the invasive species will share the same habitat areas in the west. In the south, it is seen that population of *E. orbicularis* will encounter the invasive species. Currently, the *M. caspica* species does not share various habitats with other native species and the invasive species. However, since climate change is shifting the habitat areas of these species, it is predicted that the species will face much more habitat and food competition in the future. Another critical issue here is the extent to which freshwater sources, on which turtle species depend, will be affected by climate change in the future. It is predicted that freshwater resources in Türkiye will be affected by climate change. Significant decreases in groundwater levels have been observed, especially in regions where agricultural activities have increased (Yılmaz et al. 2021). Besides, along with these decreases, the decrease in rainfall and the increase in evaporation as a result of climate change have also caused a decrease in water levels in lakes (Yılmaz et al. 2021). The decrease in water levels in rivers due to water use can also cause a slowdown in water flow (Fujihara et al. 2009). This can lead to serious floods during rainy periods, resulting in damage to water quality, river structure, and the living organisms within. These data indicate that droughts will likely continue to affect freshwater and the living organisms in these habitats in the future. While many groups of organisms are expected to be negatively affected by this situation, it is also expected that invasive or highly tolerant groups will have increased survival and spread potential under these conditions. It is also important to consider barriers that prevent the distribution of species. It is estimated that various barriers are currently restricting the distribution of species, and that these barriers will further limit migration in the future as well. Increasing road networks have fragmented the landscape, making roadkill one of the greatest threats to freshwater turtles (Gibbs and Shriver 2002). Females may travel long distances during nesting excursions (Christens and Bider 1987; Joyal et al. 2001) and frequently encounter significant mortality when crossing roads (Gibbs and Shriver 2002) or when exposed to terrestrial predators (Seigel 1980). The construction of dams, particularly hydroelectric dams, poses one of the greatest threats to migration of freshwater turtles due to the fact that they sever river connections and alter the direction of water flow (Harper et al. 2021; Barcenas‐Garcia et al. 2022).

Climate change has an impact on species in terms of their behavior, physiology, abundance, and distribution (Franks and Hoffmann 2012). Changes in atmospheric CO_2_ concentration affect metabolic and developmental activities such as photosynthesis, respiration, and growth in living organisms through changes in temperature and precipitation (Hughes 2000). When resources are limited, the growth of red‐eared slider turtle populations has contributed to a decline in native turtle populations in the areas where they were introduced, as red‐eared sliders have been observed to use limited food resources more efficiently than native species to grow and develop (Pearson et al. 2015). Growth pressure has been demonstrated on the native *E. orbicularis* species by the *T. scripta* species. In the experimental environment, it has been reported that the *E. orbicularis* species experienced greater weight loss and increased mortality rates compared with the invasive species present in the same environment (Cadi and Joly 2004). Within its fundamental niche, including tolerance limits, *T. scripta* occupies a wide range of environmental conditions and currently benefits from favorable climatic conditions across much of Europe and Asia (Espindola et al. 2019). Increasing temperatures associated with climate change suggest that this niche may continue to expand.

Aside from the negative impact they have on ecosystems and biodiversity, invasive species have also been associated with a high economic cost. Approximately US$ 314 billion is estimated to be caused each year by non‐native species invasions in the six nations (Pimentel et al. 2001). Countless countries have calculated the economic cost of invasive species. Studies have shown that this cost varies depending on the group of organisms (Cuthbert et al. 2022; Haubrock et al. 2021). Therefore, controlling these species and identifying and taking precautions in potential distribution areas is important for both biodiversity and economic outcomes. In Türkiye, the cost of invasive species between 1960 and 2022 was calculated to be approximately US$ 4.1 billion (Tarkan et al. 2024). The *T. scripta* species is not included in this cost calculation. Due to the lack of comprehensive cost and impact assessments for this species, the cost calculation is unknown (Tarkan et al. 2024).

## Author Contributions

**Nilgün Kaya:** conceptualization (lead), funding acquisition (equal), methodology (lead), visualization (lead), writing – original draft (lead). **Harun İnci:** conceptualization (supporting), methodology (supporting), writing – review and editing (supporting). **İrem Şarlak:** methodology (supporting), resources (equal), writing – review and editing (supporting). **Tuğçe Yetim:** methodology (equal), resources (equal). **Ceren Nur Özgül:** methodology (supporting), resources (equal), writing – review and editing (supporting). **Oya Özuluğ:** conceptualization (supporting), supervision (equal), writing – review and editing (supporting). **Murat Tosunoğlu:** funding acquisition (equal), supervision (equal), writing – review and editing (equal).

## Conflicts of Interest

The authors declare no conflicts of interest.

## Supporting information


**Table S1:** Presence data for the species of *Trachemys scripta*, *Mauremys caspica*, *Mauremys rivulata*, and *Emys orbicularis* from various sources.

## Data Availability

The data of this study are available in Appendix S1.
