# Supplementary material for: Pressure of Invasive Alien Species Trachemys scripta on Native Species Under Future Climate Change Scenarios
Source: Ecol Evol. 2026 Feb 11;16(2):e73084. doi: 10.1002/ece3.73084 (PMC12893788; doi:10.1002/ece3.73084)
Supplement: Supplementary file 1 — Table S1: Presence data for the species of Trachemys scripta, Mauremys caspica, Mauremys rivulata, and Emys orbicularis from various sources. [file ECE3-16-e73084-s001.docx]

**Appendix A.**

**Table 1.** Presence data for the species of *Trachemys* *scripta*, *Mauremys* *caspica*, *Mauremys* *rivulata*, and *Emys* *orbicularis* from various sources.

| **Species** | **Coordinates** | **Country** | **Locality** | **Date** | **References** |
| --- | --- | --- | --- | --- | --- |
| *Trachemys scripta* | 40.143253,26.424169 | Türkiye | Çanakkale | 4.05.2023 | Field study |
| *Trachemys scripta* | 40.090669,26.363156 | Türkiye | Çanakkale | 21.05.2018 | Field study |
| *Trachemys scripta* | 40.144093,26.456388 | Türkiye | Çanakkale | 7.09.2021 | Field study |
| *Trachemys scripta* | 40.143253,26.424169 | Türkiye | Çanakkale | 8.07.2023 | Field study |
| *Trachemys scripta* | 40.132880,26.509744 | Türkiye | Çanakkale | 2023 | Field study |
| *Trachemys scripta* | 40.139319,26.482099 | Türkiye | Çanakkale | 2023 | Field study |
| *Trachemys scripta* | 41.19477,29.20504 | Türkiye | Istanbul | 15.06.2023 | Field study |
| *Trachemys scripta* | 36.081789,32.894666 | Türkiye | Mersin | 2015 | Çiçek and Ayaz, 2015 |
| *Trachemys scripta* | 36.081789,32.894666 | Türkiye | Anamur, Mersin | 2005 | Çiçek and Ayaz, 2015 |
| *Trachemys scripta* | 36.07458333,32.8772778 | Türkiye | Anamur, Mersin | 2022 | Eldeleklioğlu et al., 2023 |
| *Trachemys scripta* | 41.06961,30.70330 | Türkiye | Sakarya | 30.06.2023 | <http://www.turkherptil.org/> |
| *Trachemys scripta* | 38.48058,27.22467 | Türkiye | İzmir | 9.08.2021 | http://www.turkherptil.org/ |
| *Trachemys scripta* | 38.58675,27.04355 | Türkiye | İzmir | 9.08.2021 | http://www.turkherptil.org/ |
| *Trachemys scripta* | 37.8733,40.2734 | Türkiye | Diyarbakir | 17.02.2022 | http://www.turkherptil.org/ |
| *Trachemys scripta* | 41.85498,27.94149 | Türkiye | Kırklareli | 18.10.2020 | http://www.turkherptil.org/ |
| *Trachemys scripta* | 37.6239,29.0777 | Türkiye | Denizli | 13.05.2020 | http://www.turkherptil.org/ |
| *Trachemys scripta* | 41.59962,32.28637 | Türkiye | Bartın | 10.05.2019 | http://www.turkherptil.org/ |
| *Trachemys scripta* | 36.1371,32.7527 | Türkiye | Mersin | 6.09.2018 | http://www.turkherptil.org/ |
| *Trachemys scripta* | 41.5830,32.3641 | Türkiye | Bartın | 27.09.2018 | http://www.turkherptil.org/ |
| *Trachemys scripta* | 41.3998,36.0835 | Türkiye | Samsun | 8.03.2017 | http://www.turkherptil.org/ |
| *Trachemys scripta* | 40.8539,31.7849 | Türkiye | Bolu | 18.05.2013 | http://www.turkherptil.org/ |
| *Trachemys scripta* | 40.8002,29.9492 | Türkiye | Kocaeli | 27.04.2015 | http://www.turkherptil.org/ |
| *Trachemys scripta* | 38.6222,27.3975 | Türkiye | Manisa | 28.09.2016 | http://www.turkherptil.org/ |
| *Trachemys scripta* | 36.2721,33.5299 | Türkiye | Mersin | 2.02.2017 | http://www.turkherptil.org/ |
| *Trachemys scripta* | 37.0049,35.3322 | Türkiye | Adana | 24.09.2016 | http://www.turkherptil.org/ |
| *Trachemys scripta* | 40.7373,31.4315 | Türkiye | Bolu | 11.06.2016 | http://www.turkherptil.org/ |
| *Trachemys scripta* | 40.9446,29.1998 | Türkiye | Istanbul | 16.02.2016 | http://www.turkherptil.org/ |
| *Trachemys scripta* | 36.9106,28.2949 | Türkiye | Muğla | 25.09.2015 | http://www.turkherptil.org/ |
| *Trachemys scripta* | 39.7751,32.7471 | Türkiye | Ankara | 8.07.2015 | http://www.turkherptil.org/ |
| *Trachemys scripta* | 40.5652,31.3167 | Türkiye | Bolu | 24.09.2015 | http://www.turkherptil.org/ |
| *Trachemys scripta* | 41.27116,28.73349 | Türkiye | Istanbul | 6.07.2015 | http://www.turkherptil.org/ |
| *Trachemys scripta* | 41.17864,29.00828 | Türkiye | Istanbul | 12.07.2015 | http://www.turkherptil.org/ |
| *Trachemys scripta* | 36.9341,28.5360 | Türkiye | Muğla | 1.04.2015 | http://www.turkherptil.org/ |
| *Trachemys scripta* | 38.5144,27.1897 | Türkiye | İzmir | 2.04.2013 | http://www.turkherptil.org/ |
| *Trachemys scripta* | 40.7035,30.3263 | Türkiye | Sakarya | 21.11.2006 | http://www.turkherptil.org/ |
| *Trachemys scripta* | 41.00562,29.06657 | Türkiye | Istanbul | 6.09.2013 | http://www.turkherptil.org/ |
| *Trachemys scripta* | 41.2837,41.6918 | Türkiye | Artvin | 14.07.2012 | http://www.turkherptil.org/ |
| *Trachemys scripta* | 39.60713,26.79189 | Türkiye | Balıkesir | 16.08.2010 | http://www.turkherptil.org/ |
| *Trachemys scripta* | 37.1192,35.2726 | Türkiye | Adana | 15.08.2010 | http://www.turkherptil.org/ |
| *Trachemys scripta* | 37.6725,29.2252 | Türkiye | Denizli | 17.06.2011 | http://www.turkherptil.org/ |
| *Trachemys scripta* | 39.991349,32.651161 | Türkiye | Ankara | 8.8.2022 | https://www.gbif.org/ |
| *Trachemys scripta* | 39.869949,32.777039 | Türkiye | Ankara | 22.5.2022 | https://www.gbif.org/ |
| *Trachemys scripta* | 39.869975,32.777002 | Türkiye | Ankara | 22.5.2022 | https://www.gbif.org/ |
| *Trachemys scripta* | 39.866541,32.740413 | Türkiye | Ankara | 1.4.2022 | https://www.gbif.org/ |
| *Trachemys scripta* | 39.990843,32.64957 | Türkiye | Ankara | 25.7.2020 | https://www.gbif.org/ |
| *Trachemys scripta* | 39.992107,32.647045 | Türkiye | Ankara | 22.7.2021 | https://www.gbif.org/ |
| *Trachemys scripta* | 39.992234,32.646471 | Türkiye | Ankara | 21.8.2021 | https://www.gbif.org/ |
| *Trachemys scripta* | 39.778109,32.793909 | Türkiye | Ankara | 30.7.2018 | https://www.gbif.org/ |
| *Trachemys scripta* | 39.87078,32.740003 | Türkiye | Ankara | 21.5.2021 | https://www.gbif.org/ |
| *Trachemys scripta* | 39.869265,32.739897 | Türkiye | Ankara | 22.4.2021 | https://www.gbif.org/ |
| *Trachemys scripta* | 39.869949,32.777039 | Türkiye | Ankara | 22.5.2022 | https://www.gbif.org/ |
| *Trachemys scripta* | 41.103318,29.029511 | Türkiye | Istanbul | 22.5.2022 | https://www.gbif.org/ |
| *Trachemys scripta* | 39.869975,32.777002 | Türkiye | Ankara | 22.5.2022 | https://www.gbif.org/ |
| *Trachemys scripta* | 36.835683,30.593221 | Türkiye | Antalya | 1.3.2020 | https://www.gbif.org/ |
| *Trachemys scripta* | 36.882805,30.663771 | Türkiye | Antalya | 26.2.2023 | https://www.gbif.org/ |
| *Trachemys scripta* | 36.88279,30.66375 | Türkiye | Antalya | 26.2.2023 | https://www.gbif.org/ |
| *Trachemys scripta* | 36.999237,30.823643 | Türkiye | Antalya | 24.8.2022 | https://www.gbif.org/ |
| *Trachemys scripta* | 36.882982,30.66347 | Türkiye | Antalya | 11.5.2022 | https://www.gbif.org/ |
| *Trachemys scripta* | 36.396541,30.475473 | Türkiye | Antalya | 20.4.2022 | https://www.gbif.org/ |
| *Trachemys scripta* | 36.859836,30.729981 | Türkiye | Antalya | 2.11.2021 | https://www.gbif.org/ |
| *Trachemys scripta* | 36.859578,30.730051 | Türkiye | Antalya | 2.11.2021 | https://www.gbif.org/ |
| *Trachemys scripta* | 36.718803,31.560257 | Türkiye | Antalya | 15.10.2021 | https://www.gbif.org/ |
| *Trachemys scripta* | 36.80228,31.352203 | Türkiye | Antalya | 10.9.2019 | https://www.gbif.org/ |
| *Trachemys scripta* | 36.802208,31.35249 | Türkiye | Antalya | 10.9.2019 | https://www.gbif.org/ |
| *Trachemys scripta* | 36.801867,31.352188 | Türkiye | Antalya | 10.9.2019 | https://www.gbif.org/ |
| *Trachemys scripta* | 36.802218,31.352503 | Türkiye | Antalya | 10.9.2019 | https://www.gbif.org/ |
| *Trachemys scripta* | 39.576581,26.758064 | Türkiye | Balikesir | 16.8.2010 | https://www.gbif.org/ |
| *Trachemys scripta* | 37.913696,29.11871 | Türkiye | Denizli | 15.8.2023 | https://www.gbif.org/ |
| *Trachemys scripta* | 37.915964,40.272885 | Türkiye | Diyarbakir | 6.6.2022 | https://www.gbif.org/ |
| *Trachemys scripta* | 37.915752,40.273124 | Türkiye | Diyarbakir | 17.2.2022 | https://www.gbif.org/ |
| *Trachemys scripta* | 37.915823,40.273336 | Türkiye | Diyarbakir | 27.10.2021 | https://www.gbif.org/ |
| *Trachemys scripta* | 37.915951,40.273215 | Türkiye | Diyarbakir | 21.9.2021 | https://www.gbif.org/ |
| *Trachemys scripta* | 37.916175,40.273813 | Türkiye | Diyarbakir | 28.9.2021 | https://www.gbif.org/ |
| *Trachemys scripta* | 37.915953,40.273312 | Türkiye | Diyarbakir | 17.8.2021 | https://www.gbif.org/ |
| *Trachemys scripta* | 37.915801,40.273055 | Türkiye | Diyarbakir | 13.8.2021 | https://www.gbif.org/ |
| *Trachemys scripta* | 37.915852,40.27269 | Türkiye | Diyarbakir | 13.8.2021 | https://www.gbif.org/ |
| *Trachemys scripta* | 37.916192,40.273676 | Türkiye | Diyarbakir | 6.3.2021 | https://www.gbif.org/ |
| *Trachemys scripta* | 37.916247,40.273919 | Türkiye | Diyarbakir | 27.10.2020 | https://www.gbif.org/ |
| *Trachemys scripta* | 37.916123,40.273549 | Türkiye | Diyarbakir | 12.10.2020 | https://www.gbif.org/ |
| *Trachemys scripta* | 37.916191,40.273747 | Türkiye | Diyarbakir | 15.10.2020 | https://www.gbif.org/ |
| *Trachemys scripta* | 37.915391,40.276366 | Türkiye | Diyarbakir | 31.8.2020 | https://www.gbif.org/ |
| *Trachemys scripta* | 39.772775,30.538359 | Türkiye | Eskisehir | 21.3.2023 | https://www.gbif.org/ |
| *Trachemys scripta* | 39.774892,30.549257 | Türkiye | Eskisehir | 3.7.2022 | https://www.gbif.org/ |
| *Trachemys scripta* | 41.179324,28.958854 | Türkiye | Istanbul | 5.7.2023 | https://www.gbif.org/ |
| *Trachemys scripta* | 41.04901,29.015508 | Türkiye | Istanbul | 10.9.2023 | https://www.gbif.org/ |
| *Trachemys scripta* | 41.046328,29.016463 | Türkiye | Istanbul | 10.9.2023 | https://www.gbif.org/ |
| *Trachemys scripta* | 41.7677,28.635212 | Türkiye | Istanbul | 19.8.2023 | https://www.gbif.org/ |
| *Trachemys scripta* | 41.007661,28.635289 | Türkiye | Istanbul | 19.8.2023 | https://www.gbif.org/ |
| *Trachemys scripta* | 40.999216,28.881314 | Türkiye | Istanbul | 28.7.2023 | https://www.gbif.org/ |
| *Trachemys scripta* | 41.134634,29.032673 | Türkiye | Istanbul | 12.7.2023 | https://www.gbif.org/ |
| *Trachemys scripta* | 41.108243,29.054165 | Türkiye | Istanbul | 12.7.2023 | https://www.gbif.org/ |
| *Trachemys scripta* | 41.102411,29.030762 | Türkiye | Istanbul | 11.7.2023 | https://www.gbif.org/ |
| *Trachemys scripta* | 40.999061,28.881159 | Türkiye | Istanbul | 19.6.2023 | https://www.gbif.org/ |
| *Trachemys scripta* | 40.9967,29.117547 | Türkiye | Istanbul | 3.6.2023 | https://www.gbif.org/ |
| *Trachemys scripta* | 41.131396,29.036007 | Türkiye | Istanbul | 9.6.2023 | https://www.gbif.org/ |
| *Trachemys scripta* | 41.140001,29.030414 | Türkiye | Istanbul | 15.5.2023 | https://www.gbif.org/ |
| *Trachemys scripta* | 41.140001,29.030414 | Türkiye | Istanbul | 15.5.2023 | https://www.gbif.org/ |
| *Trachemys scripta* | 40.998956,28.880342 | Türkiye | Istanbul | 26.5.2023 | https://www.gbif.org/ |
| *Trachemys scripta* | 40.99901,28.881198 | Türkiye | Istanbul | 26.5.2023 | https://www.gbif.org/ |
| *Trachemys scripta* | 41.10358,29.029873 | Türkiye | Istanbul | 22.5.2023 | https://www.gbif.org/ |
| *Trachemys scripta* | 41.103458,29.029658 | Türkiye | Istanbul | 22.5.2023 | https://www.gbif.org/ |
| *Trachemys scripta* | 41.104838,29.32702 | Türkiye | Istanbul | 22.5.2023 | https://www.gbif.org/ |
| *Trachemys scripta* | 41.102893,29.029458 | Türkiye | Istanbul | 22.5.2023 | https://www.gbif.org/ |
| *Trachemys scripta* | 41.103477,29.029759 | Türkiye | Istanbul | 22.5.2023 | https://www.gbif.org/ |
| *Trachemys scripta* | 41.391699,28.4523 | Türkiye | Istanbul | 22.4.2023 | https://www.gbif.org/ |
| *Trachemys scripta* | 41.102362,29.030821 | Türkiye | Istanbul | 4.5.2023 | https://www.gbif.org/ |
| *Trachemys scripta* | 41.008238,28.978359 | Türkiye | Istanbul | 29.4.2023 | https://www.gbif.org/ |
| *Trachemys scripta* | 41.19546,28.989409 | Türkiye | Istanbul | 23.4.2023 | https://www.gbif.org/ |
| *Trachemys scripta* | 41.184476,28.994838 | Türkiye | Istanbul | 23.4.2023 | https://www.gbif.org/ |
| *Trachemys scripta* | 41.103252,29.029647 | Türkiye | Istanbul | 14.4.2023 | https://www.gbif.org/ |
| *Trachemys scripta* | 41.131395,29.035139 | Türkiye | Istanbul | 14.4.2023 | https://www.gbif.org/ |
| *Trachemys scripta* | 41.108674,29.054276 | Türkiye | Istanbul | 9.4.2023 | https://www.gbif.org/ |
| *Trachemys scripta* | 41.102385,29.030867 | Türkiye | Istanbul | 21.3.2023 | https://www.gbif.org/ |
| *Trachemys scripta* | 41.131593,29.035832 | Türkiye | Istanbul | 26.2.2023 | https://www.gbif.org/ |
| *Trachemys scripta* | 40.997742,29.120167 | Türkiye | Istanbul | 16.8.2022 | https://www.gbif.org/ |
| *Trachemys scripta* | 41.102877,29.030721 | Türkiye | Istanbul | 3.1.2023 | https://www.gbif.org/ |
| *Trachemys scripta* | 41.108226,29.054112 | Türkiye | Istanbul | 8.5.2022 | https://www.gbif.org/ |
| *Trachemys scripta* | 40.998995,28.880195 | Türkiye | Istanbul | 7.10.2022 | https://www.gbif.org/ |
| *Trachemys scripta* | 41.194203,28.9894 | Türkiye | Istanbul | 11.9.2022 | https://www.gbif.org/ |
| *Trachemys scripta* | 41.048242,29.015719 | Türkiye | Istanbul | 19.8.2022 | https://www.gbif.org/ |
| *Trachemys scripta* | 41.04972,29.014772 | Türkiye | Istanbul | 19.8.2022 | https://www.gbif.org/ |
| *Trachemys scripta* | 40.996718,29.119301 | Türkiye | Istanbul | 4.6.2022 | https://www.gbif.org/ |
| *Trachemys scripta* | 41.102605,29.030658 | Türkiye | Istanbul | 11.8.2022 | https://www.gbif.org/ |
| *Trachemys scripta* | 41.131439,29.035742 | Türkiye | Istanbul | 19.8.2022 | https://www.gbif.org/ |
| *Trachemys scripta* | 41.105804,29.056705 | Türkiye | Istanbul | 3.8.2022 | https://www.gbif.org/ |
| *Trachemys scripta* | 41.107748,29.051084 | Türkiye | Istanbul | 26.3.2022 | https://www.gbif.org/ |
| *Trachemys scripta* | 41.122176,29.031477 | Türkiye | Istanbul | 7.6.2022 | https://www.gbif.org/ |
| *Trachemys scripta* | 40.999519,28.881201 | Türkiye | Istanbul | 22.6.2022 | https://www.gbif.org/ |
| *Trachemys scripta* | 41.04906,29.015395 | Türkiye | Istanbul | 22.5.2022 | https://www.gbif.org/ |
| *Trachemys scripta* | 41.048996,29.015444 | Türkiye | Istanbul | 22.5.2022 | https://www.gbif.org/ |
| *Trachemys scripta* | 41.102365,29.030868 | Türkiye | Istanbul | 22.5.2022 | https://www.gbif.org/ |
| *Trachemys scripta* | 41.10338,29.29849 | Türkiye | Istanbul | 22.5.2022 | https://www.gbif.org/ |
| *Trachemys scripta* | 41.102542,29.030728 | Türkiye | Istanbul | 23.5.2022 | https://www.gbif.org/ |
| *Trachemys scripta* | 41.103711,29.030395 | Türkiye | Istanbul | 22.5.2022 | [https://www.gbif.org/](http://www.turkherptil.org/) |
| *Trachemys scripta* | 41.103536,29.029523 | Türkiye | Istanbul | 14.4.2021 | [https://www.gbif.org/](http://www.turkherptil.org/) |
| *Trachemys scripta* | 41.103021,29.029609 | Türkiye | Istanbul | 22.5.2022 | [https://www.gbif.org/](http://www.turkherptil.org/) |
| *Trachemys scripta* | 41.103536,29.029523 | Türkiye | Istanbul | 10.6.2021 | [https://www.gbif.org/](http://www.turkherptil.org/) |
| *Trachemys scripta* | 41.107704,29.05112 | Türkiye | Istanbul | 8.5.2022 | [https://www.gbif.org/](http://www.turkherptil.org/) |
| *Trachemys scripta* | 41.108204,29.054324 | Türkiye | Istanbul | 8.5.2022 | [https://www.gbif.org/](http://www.turkherptil.org/) |
| *Trachemys scripta* | 41.107767,29.051154 | Türkiye | Istanbul | 8.5.2022 | [https://www.gbif.org/](http://www.turkherptil.org/) |
| *Trachemys scripta* | 41.016083,29.048406 | Türkiye | Istanbul | 30.4.2022 | [https://www.gbif.org/](http://www.turkherptil.org/) |
| *Trachemys scripta* | 41.107654,29.050995 | Türkiye | Istanbul | 8.5.2022 | [https://www.gbif.org/](http://www.turkherptil.org/) |
| *Trachemys scripta* | 41.003944,28.981779 | Türkiye | Istanbul | 10.5.2022 | [https://www.gbif.org/](http://www.turkherptil.org/) |
| *Trachemys scripta* | 41.107646,29.051003 | Türkiye | Istanbul | 8.5.2022 | [https://www.gbif.org/](http://www.turkherptil.org/) |
| *Trachemys scripta* | 41.107767,29.051064 | Türkiye | Istanbul | 8.5.2022 | [https://www.gbif.org/](http://www.turkherptil.org/) |
| *Trachemys scripta* | 41.107554,29.05091 | Türkiye | Istanbul | 8.5.2022 | [https://www.gbif.org/](http://www.turkherptil.org/) |
| *Trachemys scripta* | 41.015876,29.048299 | Türkiye | Istanbul | 30.4.2022 | [https://www.gbif.org/](http://www.turkherptil.org/) |
| *Trachemys scripta* | 41.107527,29.051165 | Türkiye | Istanbul | 8.5.2022 | [https://www.gbif.org/](http://www.turkherptil.org/) |
| *Trachemys scripta* | 41.177591,28.983562 | Türkiye | Istanbul | 10.4.2022 | [https://www.gbif.org/](http://www.turkherptil.org/) |
| *Trachemys scripta* | 41.131098,29.035699 | Türkiye | Istanbul | 2.4.2022 | [https://www.gbif.org/](http://www.turkherptil.org/) |
| *Trachemys scripta* | 40.99707,29.118513 | Türkiye | Istanbul | 25.06.2018 | [https://www.gbif.org/](http://www.turkherptil.org/) |
| *Trachemys scripta* | 38.231116,27.994582 | Türkiye | Izmir | 22.9.2022 | [https://www.gbif.org/](http://www.turkherptil.org/) |
| *Trachemys scripta* | 37.949638,27.363924 | Türkiye | Izmir | 22.4.2016 | [https://www.gbif.org/](http://www.turkherptil.org/) |
| *Trachemys scripta* | 39.118953,27.164942 | Türkiye | Izmir | 9.8.2018 | [https://www.gbif.org/](http://www.turkherptil.org/) |
| *Trachemys scripta* | 38.512388,27.230606 | Türkiye | Izmir | 10.8.2021 | [https://www.gbif.org/](http://www.turkherptil.org/) |
| *Trachemys scripta* | 41.17738,28.980936 | Türkiye | Istanbul | 7.9.2010 | [https://www.gbif.org/](http://www.turkherptil.org/) |
| *Trachemys scripta* | 39.48124,29.90068 | Türkiye | Kütahya | 20.10.2022 | [https://www.gbif.org/](http://www.turkherptil.org/) |
| *Trachemys scripta* | 40.893886,29.375586 | Türkiye | Kocaeli | 23.9.2022 | [https://www.gbif.org/](http://www.turkherptil.org/) |
| *Trachemys scripta* | 40.779385,29.423673 | Türkiye | Kocaeli | 23.7.2022 | [https://www.gbif.org/](http://www.turkherptil.org/) |
| *Trachemys scripta* | 38.604008,27.383948 | Türkiye | Manisa | 13.7.2022 | [https://www.gbif.org/](http://www.turkherptil.org/) |
| *Trachemys scripta* | 38.602566,27.386692 | Türkiye | Manisa | 13.7.2022 | [https://www.gbif.org/](http://www.turkherptil.org/) |
| *Trachemys scripta* | 36.77286,32.832889 | Türkiye | Mersin | 11.7.2010 | [https://www.gbif.org/](http://www.turkherptil.org/) |
| *Trachemys scripta* | 36.81572,32.895381 | Türkiye | Mersin | 15.7.2021 | https://www.gbif.org/ |
| *Trachemys scripta* | 36.081641,32.895447 | Türkiye | Mersin | 4.5.2015 | https://www.gbif.org/ |
| *Trachemys scripta* | 36.968796,28.271988 | Türkiye | Muğla | 7.6.2019 | https://www.gbif.org/ |
| *Trachemys scripta* | 36.814603,28.635972 | Türkiye | Muğla | 1.10.2022 | https://www.gbif.org/ |
| *Trachemys scripta* | 36.663393,29.112239 | Türkiye | Muğla | 13.1.2016 | https://www.gbif.org/ |
| *Trachemys scripta* | 38.24539,35.200843 | Türkiye | Nigde | 26.7.2022 | https://www.gbif.org/ |
| *Trachemys scripta* | 37.066569,36.233471 | Türkiye | Osmaniye | 17.5.2017 | https://www.gbif.org/ |
| *Trachemys scripta* | 41.138287,27.883141 | Türkiye | Tekirdag | 24.6.2020 | https://www.gbif.org/ |
| *Trachemys scripta* | 36.081554,32.895592 | Türkiye | Mersin | 1.10.2016 | https://www.gbif.org/ |
| *Trachemys scripta* | 40.2201,29.040387 | Türkiye | Bursa | 13.9.2019 | https://www.gbif.org/ |
| *Trachemys scripta* | 40.98284,39.60942 | Türkiye | Trabzon | 17.10.2020 | Koç-Gür et al., 2021 |
| *Trachemys scripta* | 39.8989,32.4521 | Türkiye | Ankara | 2005-2009 | Şahin, 2021 |
| *Trachemys scripta* | 40.145226,26.418724 | Türkiye | Çanakkale | 2014 | Uysal et al., 2018 |
| *Trachemys scripta* | 40.101712,26.377010 | Türkiye | Çanakkale | 2014 | Uysal et al., 2018 |
| *Trachemys scripta* | 40.239942,27.239655 | Türkiye | Çanakkale | 2014 | Uysal et al., 2018 |
| *Trachemys scripta* | 40.623450,26.885930 | Türkiye | Çanakkale | 2014 | Uysal et al., 2018 |
| *Trachemys scripta* | 36.700726,27.675086 | Türkiye | Muğla | 2017-2019 | Yakin et al., 2024 |
| *Emys orbicularis* | 41.625329,26.633957 | Türkiye | Edirne | 17.09.2009 | Field study |
| *Emys orbicularis* | 40.773310,26.236823 | Türkiye | Edirne | 26.09.2016 | Field study |
| *Emys orbicularis* | 40.740933,26.127317 | Türkiye | Edirne | 15.08.2007 | Field study |
| *Emys orbicularis* | 40.779918,26.241524 | Türkiye | Edirne | 15.08.2007 | Field study |
| *Emys orbicularis* | 40.781480,26.238081 | Türkiye | Edirne | 22.06.2008 | Field study |
| *Emys orbicularis* | 41.193805,26.733050 | Türkiye | Edirne | 27.03.2006 | Field study |
| *Emys orbicularis* | 41.480085,26.685513 | Türkiye | Edirne | 4.03.2014 | Field study |
| *Emys orbicularis* | 41.944786,26.701650 | Türkiye | Edirne | 9.05.2014 | Field study |
| *Emys orbicularis* | 41.826678,26.785350 | Türkiye | Edirne | 1.06.2014 | Field study |
| *Emys orbicularis* | 41.844411,26.700454 | Türkiye | Edirne | 13.08.2014 | Field study |
| *Emys orbicularis* | 41.624600,26.633981 | Türkiye | Edirne | 27.06.2014 | Field study |
| *Emys orbicularis* | 40.740231,26.127369 | Türkiye | Edirne | 17.09.2009 | Field study |
| *Emys orbicularis* | 40.779211,26.241569 | Türkiye | Edirne | 15.08.2007 | Field study |
| *Emys orbicularis* | 41.193092,26.733069 | Türkiye | Edirne | 15.08.2007 | Field study |
| *Emys orbicularis* | 40.780778,26.238122 | Türkiye | Edirne | 27.03.2006 | Field study |
| *Emys orbicularis* | 41.479358,26.685528 | Türkiye | Edirne | 5.04.2012 | Field study |
| *Emys orbicularis* | 41.944050,26.701664 | Türkiye | Edirne | 22.06.2008 | Field study |
| *Emys orbicularis* | 41.825944,26.785361 | Türkiye | Edirne | 4.03.2014 | Field study |
| *Emys orbicularis* | 41.948486,26.867133 | Türkiye | Edirne | 9.05.2014 | Field study |
| *Emys orbicularis* | 41.212211,26.743208 | Türkiye | Edirne | 1.06.2014 | Field study |
| *Emys orbicularis* | 41.843672,26.700467 | Türkiye | Edirne | 27.06.2014 | Field study |
| *Emys orbicularis* | 41.971039,26.870194 | Türkiye | Edirne | 14.08.2014 | Field study |
| *Emys orbicularis* | 40.773310,26.236823 | Türkiye | Edirne | 26.09.2016 | Field study |
| *Emys orbicularis* | 41.426350,27.364923 | Türkiye | Kırklareli | 11.07.2020 | Field study |
| *Emys orbicularis* | 41.892441,27.999356 | Türkiye | Kırklareli | 9.04.2021 | Field study |
| *Emys orbicularis* | 41.972070,27.127260 | Türkiye | Kırklareli | 9.04.2021 | Field study |
| *Emys orbicularis* | 39.059999,34.517811 | Türkiye | Kırşehir | 17.08.2016 | Field study |
| *Emys orbicularis* | 38.770448,33.648270 | Türkiye | Ankara |  | Field study |
| *Emys orbicularis* | 38.812058,33.521558 | Türkiye | Ankara |  | Field study |
| *Emys orbicularis* | 38.924150,33.455273 | Türkiye | Ankara |  | Field study |
| *Emys orbicularis* | 38.659661,33.122039 | Türkiye | Konya |  | Field study |
| *Emys orbicularis* | 38.525233,33.246181 | Türkiye | Konya |  | Field study |
| *Emys orbicularis* | 38.298140,33.526023 | Türkiye | Aksaray |  | Field study |
| *Emys orbicularis* | 40.797127,32.127222 | Türkiye | Bolu | 2.06.2017 | Field study |
| *Emys orbicularis* | 40.845922,32.445617 | Türkiye | Bolu | 2.06.2017 | Field study |
| *Emys orbicularis* | 40.607066,31.293955 | Türkiye | Bolu | 28.06.2017 | Field study |
| *Emys orbicularis* | 41.479358,26.685528 | Türkiye | Edirne | 4.03.2014 | Field study |
| *Emys orbicularis* | 41.944050,26.701664 | Türkiye | Edirne | 9.05.2014 | Field study |
| *Emys orbicularis* | 41.343672,26.700467 | Türkiye | Edirne | 13.08.2014 | Field study |
| *Emys orbicularis* | 40.136796,25.775517 | Türkiye | Çanakkale | 4.07.2019 | Museum Collection |
| *Emys orbicularis* | 42.022483,34.909871 | Türkiye | Sinop | 20.06.2009 | Museum Collection |
| *Emys orbicularis* | 39.860300,36.527657 | Türkiye | Sivas | 27.04.2014 | Museum Collection |
| *Emys orbicularis* | 38.34,33.97 | Türkiye | Aksaray | 15.06.2008 | https://www.gbif.org/ |
| *Emys orbicularis* | 39.945222,32.967325 | Türkiye | Ankara | 25.06.2019 | https://www.gbif.org/ |
| *Emys orbicularis* | 36.816433,31.296314 | Türkiye | Antalya | 18.05.2016 | https://www.gbif.org/ |
| *Emys orbicularis* | 36.771023,30.488989 | Türkiye | Antalya | 15.09.2023 | https://www.gbif.org/ |
| *Emys orbicularis* | 37.023573,31.803293 | Türkiye | Antalya | 15.04.2012 | https://www.gbif.org/ |
| *Emys orbicularis* | 36.834213,31.3232 | Türkiye | Antalya, Çolaklı | 18.05.2016 | https://www.gbif.org/ |
| *Emys orbicularis* | 37.594646,27.446594 | Türkiye | Azap Lake | 20.04.2009 | https://www.gbif.org/ |
| *Emys orbicularis* | 37.541309,27.413635 | Türkiye | Bafa Lake | 28.10.1989 | https://www.gbif.org/ |
| *Emys orbicularis* | 40.48,27.97 | Türkiye | Balıkesir | 4.07.2014 | https://www.gbif.org/ |
| *Emys orbicularis* | 37.636799,31.572475 | Türkiye | Beysehir Lake | 18.05.1982 | https://www.gbif.org/ |
| *Emys orbicularis* | 40.398624,28.984792 | Türkiye | Bursa | 24.07.2023 | https://www.gbif.org/ |
| *Emys orbicularis* | 40.959456,33.690917 | Türkiye | Çankırı | 31.07.2022 | https://www.gbif.org/ |
| *Emys orbicularis* | 36.91,28.69 | Türkiye | Dalyan | 1.06.2009 | https://www.gbif.org/ |
| *Emys orbicularis* | 36.82,28.62 | Türkiye | Dalyan | 26.04.2005 | https://www.gbif.org/ |
| *Emys orbicularis* | 40.75,26.216667 | Türkiye | Edirne | 04.2016 | https://www.gbif.org/ |
| *Emys orbicularis* | 38.141579,30.785065 | Türkiye | Hoyran Lake | 23.05.1982 | https://www.gbif.org/ |
| *Emys orbicularis* | 41.218358,29.376995 | Türkiye | Istanbul | 3.04.2007 | https://www.gbif.org/ |
| *Emys orbicularis* | 41.315759,29.244321 | Türkiye | Istanbul | 23.04.2007 | https://www.gbif.org/ |
| *Emys orbicularis* | 38.239636,35.211437 | Türkiye | Istanbul | 11.05.2023 | https://www.gbif.org/ |
| *Emys orbicularis* | 41.210115,28.531588 | Türkiye | Istanbul | 22.04.2023 | https://www.gbif.org/ |
| *Emys orbicularis* | 41.251501,28.413828 | Türkiye | Istanbul | 1.05.2023 | https://www.gbif.org/ |
| *Emys orbicularis* | 41.179238,28.787819 | Türkiye | Istanbul | 15.01.2023 | https://www.gbif.org/ |
| *Emys orbicularis* | 40.957428,28.616252 | Türkiye | Istanbul | 26.04.2022 | https://www.gbif.org/ |
| *Emys orbicularis* | 38.004337,27.192949 | Türkiye | İzmir | 2.04.2011 | https://www.gbif.org/ |
| *Emys orbicularis* | 38.25,27.16 | Türkiye | İzmir | 17.05.2022 | https://www.gbif.org/ |
| *Emys orbicularis* | 38.432613,30.857162 | Türkiye | Karamik Swamp |  | https://www.gbif.org/ |
| *Emys orbicularis* | 38.374945,35.214515 | Türkiye | Kayseri | 14.05.2022 | https://www.gbif.org/ |
| *Emys orbicularis* | 38.296288,35.212231 | Türkiye | Kayseri | 1.05.2006 | https://www.gbif.org/ |
| *Emys orbicularis* | 38.223995,35.24288 | Türkiye | Kayseri | 14.05.2022 | https://www.gbif.org/ |
| *Emys orbicularis* | 38.296288,35.212231 | Türkiye | Kayseri | 1.05.2006 | https://www.gbif.org/ |
| *Emys orbicularis* | 38.278877,35.369026 | Türkiye | Kayseri | 14.05.2022 | https://www.gbif.org/ |
| *Emys orbicularis* | 41.687795,26.808624 | Türkiye | Kırklareli | 10.09.2020 | https://www.gbif.org/ |
| *Emys orbicularis* | 41.630721,28.125278 | Türkiye | Kırklareli | 15.04.2022 | https://www.gbif.org/ |
| *Emys orbicularis* | 41.730492,28.152752 | Türkiye | Kırklareli | 22.04.2023 | https://www.gbif.org/ |
| *Emys orbicularis* | 41.678408,26.825339 | Türkiye | Kırklareli | 10.09.2020 | https://www.gbif.org/ |
| *Emys orbicularis* | 36.283612,33.924398 | Türkiye | Mersin | 23.05.1991 | https://www.gbif.org/ |
| *Emys orbicularis* | 36.375461,33.885304 | Türkiye | Mersin | 11.04.1991 | https://www.gbif.org/ |
| *Emys orbicularis* | 36.127763,32.962903 | Türkiye | Mersin | 4.05.2015 | https://www.gbif.org/ |
| *Emys orbicularis* | 36.081783,32.895233 | Türkiye | Mersin | 10.06.2000 | https://www.gbif.org/ |
| *Emys orbicularis* | 37.530624,27.275791 | Türkiye | Muğla, Milas | 29.04.1988 | https://www.gbif.org/ |
| *Emys orbicularis* | 36.886987,28.609654 | Türkiye | Muğla | 4.03.2020 | https://www.gbif.org/ |
| *Emys orbicularis* | 38.341049,34.456139 | Türkiye | Narligöl | 29.03.2006 | https://www.gbif.org/ |
| *Emys orbicularis* | 38.354189,34.498289 | Türkiye | Niğde | 29.03.2006 | https://www.gbif.org/ |
| *Emys orbicularis* | 38.217347,35.243071 | Türkiye | Niğde | 26.07.2022 | https://www.gbif.org/ |
| *Emys orbicularis* | 38.202079,35.38851 | Türkiye | Niğde | 8.05.2023 | https://www.gbif.org/ |
| *Emys orbicularis* | 38.235056,35.015375 | Türkiye | Niğde | 8.05.2023 | https://www.gbif.org/ |
| *Emys orbicularis* | 38.220433,35.293182 | Türkiye | Niğde | 24.05.2007 | https://www.gbif.org/ |
| *Emys orbicularis* | 40.694133,30.677842 | Türkiye | Sakarya | 14.08.2000 | https://www.gbif.org/ |
| *Emys orbicularis* | 41.719114,36.017471 | Türkiye | Samsun | 10.10.2014 | https://www.gbif.org/ |
| *Emys orbicularis* | 41.063398,37.083586 | Türkiye | Samsun | 14.08.2023 | https://www.gbif.org/ |
| *Emys orbicularis* | 41.600553,36.070951 | Türkiye | Samsun | 10.10.2014 | https://www.gbif.org/ |
| *Emys orbicularis* | 41.321063,36.025449 | Türkiye | Samsun | 6.06.2019 | https://www.gbif.org/ |
| *Emys orbicularis* | 41.778606,36.151024 | Türkiye | Samsun | 28.05.2021 | https://www.gbif.org/ |
| *Emys orbicularis* | 41.280472,36.346196 | Türkiye | Samsun | 17.10.2020 | https://www.gbif.org/ |
| *Emys orbicularis* | 37.948772,27.364309 | Türkiye | İzmir, Selcuk | 2.04.2011 | https://www.gbif.org/ |
| *Emys orbicularis* | 42.105398,35.18432 | Türkiye | Sinop | 7.08.1989 | https://www.gbif.org/ |
| *Emys orbicularis* | 42.190725,34.883063 | Türkiye | Sinop | 6.08.2022 | https://www.gbif.org/ |
| *Emys orbicularis* | 42.191613,34.89421 | Türkiye | Sinop | 6.08.2022 | https://www.gbif.org/ |
| *Emys orbicularis* | 38.38,35.25 | Türkiye | Sultansazligi | 4.05.2012 | https://www.gbif.org/ |
| *Emys orbicularis* | 38.41,35.28 | Türkiye | Sultansazligi | 30.04.2013 | https://www.gbif.org/ |
| *Emys orbicularis* | 38.24,35.19 | Türkiye | Sultansazligi | 30.04.2013 | https://www.gbif.org/ |
| *Emys orbicularis* | 41.057442,27.822023 | Türkiye | Tekirdağ | 26.06.2020 | https://www.gbif.org/ |
| *Emys orbicularis* | 40.450376,28.927945 | Türkiye | Yalova | 10.08.2023 | https://www.gbif.org/ |
| *Emys orbicularis* | 38.5514,30.9531 | Türkiye | Afyonkarahisar | 3.05.2023 | https://www.gbif.org/ |
| *Emys orbicularis* | 38.6423,30.1461 | Türkiye | Afyonkarahisar | 16.06.2009 | https://www.gbif.org/ |
| *Emys orbicularis* | 38.7012,30.3577 | Türkiye | Afyonkarahisar | 12.06.2009 | https://www.gbif.org/ |
| *Emys orbicularis* | 38.6187,30.3043 | Türkiye | Afyonkarahisar | 23.06.2009 | https://www.gbif.org/ |
| *Emys orbicularis* | 37.8290,29.8935 | Türkiye | Afyonkarahisar | 21.04.2008 | https://www.gbif.org/ |
| *Emys orbicularis* | 38.6686,30.4278 | Türkiye | Afyonkarahisar | 23.06.2009 | https://www.gbif.org/ |
| *Emys orbicularis* | 39.8354,32.9685 | Türkiye | Ankara | 13.05.2017 | https://www.gbif.org/ |
| *Emys orbicularis* | 39.7285,32.7874 | Türkiye | Ankara | 31.05.2014 | https://www.gbif.org/ |
| *Emys orbicularis* | 39.7466,32.7902 | Türkiye | Ankara | 11.07.2014 | https://www.gbif.org/ |
| *Emys orbicularis* | 39.9655,32.6230 | Türkiye | Ankara | 10.10.2011 | https://www.gbif.org/ |
| *Emys orbicularis* | 40.4936,32.5119 | Türkiye | Ankara | 23.04.2012 | https://www.gbif.org/ |
| *Emys orbicularis* | 37.7887,27.3038 | Türkiye | Aydın | 7.06.2010 | https://www.gbif.org/ |
| *Emys orbicularis* | 39.62304,26.95985 | Türkiye | Balıkesir | 8.02.2017 | https://www.gbif.org/ |
| *Emys orbicularis* | 39.58903,26.91752 | Türkiye | Balıkesir | 14.05.2016 | https://www.gbif.org/ |
| *Emys orbicularis* | 39.5446,27.1604 | Türkiye | Balıkesir | 6.09.2012 | https://www.gbif.org/ |
| *Emys orbicularis* | 40.2632,27.9754 | Türkiye | Balıkesir | 24.02.2010 | https://www.gbif.org/ |
| *Emys orbicularis* | 39.7080,28.3515 | Türkiye | Balıkesir | 9.06.2013 | https://www.gbif.org/ |
| *Emys orbicularis* | 40.0870,27.5772 | Türkiye | Balıkesir | 4.07.2011 | https://www.gbif.org/ |
| *Emys orbicularis* | 40.7829,31.6795 | Türkiye | Bolu | 16.08.2022 | https://www.gbif.org/ |
| *Emys orbicularis* | 40.7638,32.0269 | Türkiye | Bolu | 14.06.2015 | https://www.gbif.org/ |
| *Emys orbicularis* | 40.6750,31.4773 | Türkiye | Bolu | 27.04.2015 | https://www.gbif.org/ |
| *Emys orbicularis* | 40.7226,31.4422 | Türkiye | Bolu | 13.07.2013 | https://www.gbif.org/ |
| *Emys orbicularis* | 40.8156,31.7976 | Türkiye | Bolu | 7.05.2011 | https://www.gbif.org/ |
| *Emys orbicularis* | 37.0656,29.7380 | Türkiye | Burdur | 18.04.2013 | https://www.gbif.org/ |
| *Emys orbicularis* | 40.1993,28.7885 | Türkiye | Bursa | 23.07.2023 | https://www.gbif.org/ |
| *Emys orbicularis* | 40.4485,29.4048 | Türkiye | Bursa | 17.05.2021 | https://www.gbif.org/ |
| *Emys orbicularis* | 40.1629,29.0964 | Türkiye | Bursa | 4.04.2017 | https://www.gbif.org/ |
| *Emys orbicularis* | 40.0033,28.5267 | Türkiye | Bursa | 15.07.2012 | https://www.gbif.org/ |
| *Emys orbicularis* | 39.8132,26.8120 | Türkiye | Çanakkale | 8.04.2017 | https://www.gbif.org/ |
| *Emys orbicularis* | 38.2175,29.8518 | Türkiye | Denizli | 13.04.2013 | https://www.gbif.org/ |
| *Emys orbicularis* | 37.6936,29.4920 | Türkiye | Denizli | 20.07.2012 | https://www.gbif.org/ |
| *Emys orbicularis* | 37.3876,28.7647 | Türkiye | Denizli | 6.05.2012 | https://www.gbif.org/ |
| *Emys orbicularis* | 37.81199,29.78108 | Türkiye | Denizli | 16.05.2009 | https://www.gbif.org/ |
| *Emys orbicularis* | 40.83532,26.64273 | Türkiye | Edirne | 22.06.2014 | https://www.gbif.org/ |
| *Emys orbicularis* | 41.2513,26.7152 | Türkiye | Edirne | 5.04.2013 | https://www.gbif.org/ |
| *Emys orbicularis* | 40.7136,26.1942 | Türkiye | Edirne | 11.07.2008 | https://www.gbif.org/ |
| *Emys orbicularis* | 39.4138,31.4783 | Türkiye | Eskişehir | 20.05.2013 | https://www.gbif.org/ |
| *Emys orbicularis* | 38.0732,30.7941 | Türkiye | Isparta | 4.05.2013 | https://www.gbif.org/ |
| *Emys orbicularis* | 38.2695,30.8954 | Türkiye | Isparta | 23.04.2012 | https://www.gbif.org/ |
| *Emys orbicularis* | 37.9789,30.8650 | Türkiye | Isparta | 1.07.2011 | https://www.gbif.org/ |
| *Emys orbicularis* | 41.19477,29.20504 | Türkiye | Istanbul | 30.03.2013 | https://www.gbif.org/ |
| *Emys orbicularis* | 41.08340,28.51528 | Türkiye | Istanbul | 31.03.2013 | https://www.gbif.org/ |
| *Emys orbicularis* | 41.17395,29.01421 | Türkiye | Istanbul | 16.09.2012 | https://www.gbif.org/ |
| *Emys orbicularis* | 41.32991,28.58675 | Türkiye | Istanbul | 14.05.2011 | https://www.gbif.org/ |
| *Emys orbicularis* | 41.19312,28.80751 | Türkiye | Istanbul | 1.07.2011 | https://www.gbif.org/ |
| *Emys orbicularis* | 38.1416,35.5276 | Türkiye | Kayseri | 19.05.2019 | https://www.gbif.org/ |
| *Emys orbicularis* | 38.32662,35.30687 | Türkiye | Kayseri | 24.05.2018 | https://www.gbif.org/ |
| *Emys orbicularis* | 38.55925,35.25986 | Türkiye | Kayseri | 20.06.2018 | https://www.gbif.org/ |
| *Emys orbicularis* | 39.6074,33.5739 | Türkiye | Kırıkkale | 21.04.2016 | https://www.gbif.org/ |
| *Emys orbicularis* | 41.74904,28.01643 | Türkiye | Kırklareli | 1.05.2023 | https://www.gbif.org/ |
| *Emys orbicularis* | 41.862672,27.955542 | Türkiye | Kırklareli | 18.10.2020 | https://www.gbif.org/ |
| *Emys orbicularis* | 41.85200,27.93108 | Türkiye | Kırklareli | 21.05.2011 | https://www.gbif.org/ |
| *Emys orbicularis* | 40.76940,29.93747 | Türkiye | Kocaeli | 13.05.2015 | https://www.gbif.org/ |
| *Emys orbicularis* | 41.0935,30.1581 | Türkiye | Kocaeli | 22.05.2014 | https://www.gbif.org/ |
| *Emys orbicularis* | 39.0280,32.7638 | Türkiye | Konya | 8.06.2012 | https://www.gbif.org/ |
| *Emys orbicularis* | 37.4806,31.7532 | Türkiye | Konya | 29.04.2010 | https://www.gbif.org/ |
| *Emys orbicularis* | 38.5128,27.7065 | Türkiye | Manisa | 25.04.2012 | https://www.gbif.org/ |
| *Emys orbicularis* | 36.80094,28.20605 | Türkiye | Muğla | 10.06.2020 | https://www.gbif.org/ |
| *Emys orbicularis* | 38.1256,35.0413 | Türkiye | Niğde | 22.05.2014 | https://www.gbif.org/ |
| *Emys orbicularis* | 37.7436,34.6628 | Türkiye | Niğde | 29.04.2010 | https://www.gbif.org/ |
| *Emys orbicularis* | 41.0265,37.7486 | Türkiye | Ordu | 21.03.2013 | https://www.gbif.org/ |
| *Emys orbicularis* | 41.1073,41.1984 | Türkiye | Rize | 8.06.2013 | https://www.gbif.org/ |
| *Emys orbicularis* | 41.07733,30.63067 | Türkiye | Sakarya | 25.06.2023 | https://www.gbif.org/ |
| *Emys orbicularis* | 41.10118,29.98318 | Türkiye | Sakarya | 30.07.2019 | https://www.gbif.org/ |
| *Emys orbicularis* | 40.7410,30.3773 | Türkiye | Sakarya | 10.07.2019 | https://www.gbif.org/ |
| *Emys orbicularis* | 41.5806,36.0280 | Türkiye | Samsun | 17.05.2016 | https://www.gbif.org/ |
| *Emys orbicularis* | 41.9740,34.9413 | Türkiye | Sinop | 19.06.2014 | https://www.gbif.org/ |
| *Emys orbicularis* | 41.9182,34.8019 | Türkiye | Sinop | 7.06.2008 | https://www.gbif.org/ |
| *Emys orbicularis* | 41.9134,34.9341 | Türkiye | Sinop | 17.05.2009 | https://www.gbif.org/ |
| *Emys orbicularis* | 40.93177,39.94869 | Türkiye | Trabzon | 26.04.2016 | https://www.gbif.org/ |
| *Emys orbicularis* | 40.9385,39.7034 | Türkiye | Trabzon | 5.04.2012 | https://www.gbif.org/ |
| *Emys orbicularis* | 41.3676,32.0803 | Türkiye | Zonguldak | 13.05.2017 | https://www.gbif.org/ |
| *Emys orbicularis* | 38.565035, 27.532617 | Türkiye | Sülüklü Göl, Manisa | 2010 | Çiçek and Ayaz, 2011 |
| *Emys orbicularis* | 39.8989,32.4521 | Türkiye | Ankara | 2005-2009 | Şahin, 2021 |
| *Emys orbicularis* | 37.7419,33.4591 | Türkiye | Karapınar, Konya | 2001-2002 | Ayaz and Budak, 2007 |
| *Emys orbicularis* | 38.5732,30.9573 | Türkiye | Pazarağaç, Afyonkarahisar | 2001-2002 | Ayaz and Budak, 2007 |
| *Emys orbicularis* | 38.7952,30.5206 | Türkiye | Çakırköy, Afyonkarahisar | 2001-2002 | Ayaz and Budak, 2007 |
| *Emys orbicularis* | 38.64813,31.18905 | Türkiye | Eber Lake, Afyonkarahisar | 2001-2002 | Ayaz and Budak, 2007 |
| *Emys orbicularis* | 38.52276,31.39769 | Türkiye | Akşehir Lake, Konya-Afyonkarahisar | 2001-2002 | Ayaz and Budak, 2007 |
| *Emys orbicularis* | 38.05275,30.89436 | Türkiye | Eğirdir Lake, Isparta | 2001-2002 | Ayaz and Budak, 2007 |
| *Emys orbicularis* | 37.63316,30.88048 | Türkiye | Kovada Lake, Isparta | 2001-2002 | Ayaz and Budak, 2007 |
| *Emys orbicularis* | 37.49900,35.91975 | Türkiye | Kozan, Adana | 7.05.2014 | Sarikaya et al., 2017 |
| *Emys orbicularis* | 36.69220,35.54873 | Türkiye | Yumurtalık, Adana | 5.02.2014 | Sarikaya et al., 2017 |
| *Emys orbicularis* | 36.05448,35.99816 | Türkiye | Samandağ, Hatay | 9.03.2016 | Yıldız et al., 2019 |
| *Emys orbicularis* | 38.05,28.766666666 | Türkiye | Buldan, Denizli | 27.06.1905 | Ayaz et al., 2008 |
| *Emys orbicularis* | 37.816666666,33.716666666 | Türkiye | Karapınar, Konya | 2010-2012 | Bayrakcı and Ayaz, 2014 |
| *Emys orbicularis* | 36.68195,28.826486111 | Türkiye | Kocagöl, Muğla | 2012-2013 | Ayaz et al., 2017 |
| *Emys orbicularis* | 37.115880555,29.599163888 | Türkiye | Gölhisar Lake, Burdur | 2012-2013 | Ayaz et al., 2017 |
| *Emys orbicularis* | 38.150894444,30.768041666 | Türkiye | Eğirdir Lake, Isparta | 2012-2013 | Ayaz et al., 2017 |
| *Emys orbicularis* | 37.325363888,32.00505 | Türkiye | Suğla, Konya | 2012-2013 | Ayaz et al., 2017 |
| *Emys orbicularis* | 36.081627777,32.894941666 | Türkiye | Anamur, Mersin | 2012-2013 | Ayaz et al., 2017 |
| *Emys orbicularis* | 36.304177777,33.951008333 | Türkiye | Akgöl, Mersin | 2012-2013 | Ayaz et al., 2017 |
| *Emys orbicularis* | 36.062611111,35.980008333 | Türkiye | Asi River, Hatay | 2012-2013 | Ayaz et al., 2017 |
| *Emys orbicularis* | 38.6,30.85 | Türkiye | Afyonkarahisar | 2005 | Ayaz et al., 2007 |
| *Emys orbicularis* | 36.791388888,28.813611111 | Türkiye | Dalaman, Muğla | 2010 | Bayrakcı et al., 2021 |
| *Emys orbicularis* | 36.055,36.034166666 | Türkiye | Tekebaşı, Hatay | 2010 | Bayrakcı et al., 2021 |
| *Emys orbicularis* | 36.610833333,33.955833333 | Türkiye | Silifke, Mersin | 2010 | Bayrakcı et al., 2021 |
| *Emys orbicularis* | 38.6026,30.467052777 | Türkiye | Afyonkarahisar | 2005 | Soylu et al., 2006 |
| *Emys orbicularis* | 38.467730555,30.884033333 | Türkiye | Karamık Lake, Afyonkarahisar | 2005 | Soylu et al., 2006 |
| *Emys orbicularis* | 38.500497222,31.300408333 | Türkiye | Akşehir Lake, Konya-Afyonkarahisar | 2005 | Soylu et al., 2006 |
| *Emys orbicularis* | 38.583713888,31.284616666 | Türkiye | Eber Lake, Afyonkarahisar | 2005 | Soylu et al., 2006 |
| *Emys orbicularis* | 37.834941666,30.884172222 | Türkiye | Eğirdir Lake, Isparta | 2005 | Soylu et al., 2006 |
| *Emys orbicularis* | 37.352308333,31.869344444 | Türkiye | Suğla Lake, Konya | 2005 | Soylu et al., 2006 |
| *Emys orbicularis* | 38.302233333,33.435694444 | Türkiye | Sultanhanı, Aksaray | 2005 | Soylu et al., 2006 |
| *Emys orbicularis* | 41.05,30.85 | Türkiye | Sakarya | 2006-2008 | Fritz et al., 2009 |
| *Emys orbicularis* | 41.283333333,36.933333333 | Türkiye | Samsun | 2006-2008 | Fritz et al., 2009 |
| *Emys orbicularis* | 41.667702777,35.802411111 | Türkiye | Samsun | 2006-2008 | Fritz et al., 2009 |
| *Emys orbicularis* | 39.450477777,29.951438888 | Türkiye | Kütahya | 2006-2008 | Fritz et al., 2009 |
| *Emys orbicularis* | 39.517669444,30.050794444 | Türkiye | Kütahya | 2006-2008 | Fritz et al., 2009 |
| *Emys orbicularis* | 39.550313888,31.750447222 | Türkiye | Eskişehir | 2006-2008 | Fritz et al., 2009 |
| *Emys orbicularis* | 39.933333333,26.233333333 | Türkiye | Çanakkale | 2006-2008 | Fritz et al., 2009 |
| *Emys orbicularis* | 40.183333333,26.233333333 | Türkiye | Uluabat Lake, Bursa | 2006-2008 | Fritz et al., 2009 |
| *Emys orbicularis* | 40.183333333,26.333333333 | Türkiye | Eceabat, Çanakkale | 2006-2008 | Fritz et al., 2009 |
| *Emys orbicularis* | 40.484219444,29.919302777 | Türkiye | Bilecik | 2006-2008 | Fritz et al., 2009 |
| *Emys orbicularis* | 40.6,26.883333333 | Türkiye | Gelibolu, Çanakkale | 2006-2008 | Fritz et al., 2009 |
| *Emys orbicularis* | 41.1,30.683333333 | Türkiye | Adapazarı | 2006-2008 | Fritz et al., 2009 |
| *Emys orbicularis* | 41.333333333,26.766666666 | Türkiye | Uzunköprü, Edirne | 2006-2008 | Fritz et al., 2009 |
| *Emys orbicularis* | 41.366666666,27.483333333 | Türkiye | Lüleburgaz, Kırklareli | 2006-2008 | Fritz et al., 2009 |
| *Emys orbicularis* | 41.45,27.516666666 | Türkiye | Kırklareli | 2006-2008 | Fritz et al., 2009 |
| *Emys orbicularis* | 38.302233333,33.435694444 | Türkiye | Konya | 2006-2008 | Fritz et al., 2009 |
| *Emys orbicularis* | 40.716819444,30.318586111 | Türkiye | Adapazarı | 2006-2008 | Fritz et al., 2009 |
| *Emys orbicularis* | 41.1,30.683333333 | Türkiye | Karasu, Adapazarı | 2006-2008 | Fritz et al., 2009 |
| *Emys orbicularis* | 41.435725,33.817633333 | Türkiye | Kastamonu | 2006-2008 | Fritz et al., 2009 |
| *Emys orbicularis* | 41.584108333,32.167416666 | Türkiye | Bartın | 2006-2008 | Fritz et al., 2009 |
| *Emys orbicularis* | 41.651122222,32.234002777 | Türkiye | Karasu, Bartın | 2006-2008 | Fritz et al., 2009 |
| *Emys orbicularis* | 38.05,28.766666666 | Türkiye | Buldan, Denizli | 2006-2008 | Fritz et al., 2009 |
| *Emys orbicularis* | 38.566666666,27.483333333 | Türkiye | Manisa | 2006-2008 | Fritz et al., 2009 |
| *Emys orbicularis* | 38.685347222,27.119075 | Türkiye | İzmir | 2006-2008 | Fritz et al., 2009 |
| *Emys orbicularis* | 37.901969444,34.502586111 | Türkiye | Niğde | 2006-2008 | Fritz et al., 2009 |
| *Emys orbicularis* | 38.302233333,33.435694444 | Türkiye | Konya | 2006-2008 | Fritz et al., 2009 |
| *Emys orbicularis* | 38.3925,35.083333333 | Türkiye | Sultanhanı, Aksaray | 2006-2008 | Fritz et al., 2009 |
| *Emys orbicularis* | 38.884775,35.719155555 | Türkiye | Kayseri | 2006-2008 | Fritz et al., 2009 |
| *Emys orbicularis* | 40.568144444,36.851291666 | Türkiye | Tokat | 2006-2008 | Fritz et al., 2009 |
| *Emys orbicularis* | 40.783333333,32.016666666 | Türkiye | Bolu | 2006-2008 | Fritz et al., 2009 |
| *Emys orbicularis* | 41.05,30.85 | Türkiye | Sakarya | 2006-2008 | Fritz et al., 2009 |
| *Emys orbicularis* | 41.435725,33.817633333 | Türkiye | Kastamonu | 2006-2008 | Fritz et al., 2009 |
| *Emys orbicularis* | 41.667702777,35.802411111 | Türkiye | Samsun | 2006-2008 | Fritz et al., 2009 |
| *Emys orbicularis* | 42.002044444,34.918791666 | Türkiye | Sinop | 2006-2008 | Fritz et al., 2009 |
| *Emys orbicularis* | 37.352308333,31.869344444 | Türkiye | Suğla Lake, Konya | 2006-2008 | Fritz et al., 2009 |
| *Emys orbicularis* | 37.468147222,33.967936111 | Türkiye | Akgöl, Konya | 2006-2008 | Fritz et al., 2009 |
| *Emys orbicularis* | 37.818186111,33.718941666 | Türkiye | Karapınar, Konya | 2006-2008 | Fritz et al., 2009 |
| *Emys orbicularis* | 37.834941666,30.884172222 | Türkiye | Eğirdir Lake, Isparta | 2006-2008 | Fritz et al., 2009 |
| *Emys orbicularis* | 37.901969444,34.502586111 | Türkiye | Niğde | 2006-2008 | Fritz et al., 2009 |
| *Emys orbicularis* | 37.916966666,34.600925000 | Türkiye | Niğde | 2006-2008 | Fritz et al., 2009 |
| *Emys orbicularis* | 38.38425,35.083333333 | Türkiye | Aksaray | 2006-2008 | Fritz et al., 2009 |
| *Emys orbicularis* | 38.384594444,35.352444444 | Türkiye | Kayseri | 2006-2008 | Fritz et al., 2009 |
| *Emys orbicularis* | 38.500497222,31.300408333 | Türkiye | Akşehir Lake, Konya-Afyonkarahisar | 2006-2008 | Fritz et al., 2009 |
| *Emys orbicularis* | 38.583713888,31.284616666 | Türkiye | Eber Lake, Afyonkarahisar | 2006-2008 | Fritz et al., 2009 |
| *Emys orbicularis* | 38.602747222,30.851169444 | Türkiye | Pazarağaç, Afyonkarahisar | 2006-2008 | Fritz et al., 2009 |
| *Emys orbicularis* | 39.718083333,30.335258333 | Türkiye | Eskişehir | 2006-2008 | Fritz et al., 2009 |
| *Emys orbicularis* | 36.1270,32.7680 | Türkiye | Anamur, Mersin | 2006-2008 | Fritz et al., 2009 |
| *Emys orbicularis* | 36.4517,33.9565 | Türkiye | Silifke, Mersin | 2006-2008 | Fritz et al., 2009 |
| *Emys orbicularis* | 36.0844,35.9882 | Türkiye | Samandağ, Hatay | 2006-2008 | Fritz et al., 2009 |
| *Emys orbicularis* | 36.2184,36.1175 | Türkiye | Asi River, Hatay | 2006-2008 | Fritz et al., 2009 |
| *Emys orbicularis* | 40.1841,33.7090 | Türkiye | Kırıkkale | 20.03.2016 | Akman et al., 2020 |
| *Emys orbicularis* | 39.9950,33.6680 | Türkiye | Kırıkkale | 25.06.2016 | Akman et al., 2020 |
| *Emys orbicularis* | 40.0013,33.8446 | Türkiye | Kırıkkale | 25.06.2016 | Akman et al., 2020 |
| *Emys orbicularis* | 39.56035,33.35832 | Türkiye | Kırıkkale | 21.08.2016 | Akman et al., 2020 |
| *Emys orbicularis* | 40.16443,25.85875 | Türkiye | Gökçeada | 2014 | Bayrakcı et al., 2016 |
| *Emys orbicularis* | 40.2,28.65 | Türkiye | Uluabat Lake, Bursa | 7.04.2006 | Ayaz and Çiçek, 2011 |
| *Emys orbicularis* | 41.5783,32.4709 | Türkiye | Bartın | 16.05.2016 | Çakmak et al., 2017 |
| *Emys orbicularis* | 41.5767,32.6522 | Türkiye | Bartın | 14.05.2016 | Çakmak et al., 2017 |
| *Emys orbicularis* | 41.5988,32.2818 | Türkiye | Bartın | 19.04.2016 | Çakmak et al., 2017 |
| *Emys orbicularis* | 41.63660,32.21794 | Türkiye | Bartın | 17.04.2016 | Çakmak et al., 2017 |
| *Mauremys rivulata* | 40.143253,26.424169 | Türkiye | Çanakkale | 15.04.2023 | Field study |
| *Mauremys rivulata* | 40.144929,26.435296 | Türkiye | Çanakkale | 17.04.2023 | Field study |
| *Mauremys rivulata* | 40.139263,26.481747 | Türkiye | Çanakkale | 4.05.2023 | Field study |
| *Mauremys rivulata* | 40.133100,26.481618 | Türkiye | Çanakkale | 3.05.2023 | Field study |
| *Mauremys rivulata* | 40.135315,26.501838 | Türkiye | Çanakkale | 3.05.2023 | Field study |
| *Mauremys rivulata* | 40.133223,26.509788 | Türkiye | Çanakkale | 30.05.2023 | Field study |
| *Mauremys rivulata* | 40.132880,26.509744 | Türkiye | Çanakkale | 31.05.2023 | Field study |
| *Mauremys rivulata* | 40.131345,26.510905 | Türkiye | Çanakkale | 3.05.2023 | Field study |
| *Mauremys rivulata* | 39.841292,26.037083 | Türkiye | Çanakkale | 6.06.2021 | Field study |
| *Mauremys rivulata* | 39.836265,26.038228 | Türkiye | Çanakkale | 6.06.2021 | Field study |
| *Mauremys rivulata* | 41.890369,26.985503 | Türkiye | Kırklareli | 26.09.2016 | Field study |
| *Mauremys rivulata* | 41.785694,27.124292 | Türkiye | Kırklareli | 26.09.2016 | Field study |
| *Mauremys rivulata* | 41.785570,27.209892 | Türkiye | Kırklareli | 10.10.2016 | Field study |
| *Mauremys rivulata* | 41.426926,27.365333 | Türkiye | Kırklareli | 10.10.2016 | Field study |
| *Mauremys rivulata* | 41.826053,27.961331 | Türkiye | Kırklareli | 10.10.2016 | Field study |
| *Mauremys rivulata* | 41.824025,26.560072 | Türkiye | Edirne | 9.05.2014 | Field study |
| *Mauremys rivulata* | 41.824025,26.560072 | Türkiye | Edirne | 22.03.2014 | Field study |
| *Mauremys rivulata* | 41.825944,26.785361 | Türkiye | Edirne | 1.06.2014 | Field study |
| *Mauremys rivulata* | 41.348172,26.683456 | Türkiye | Edirne | 19.08.2014 | Field study |
| *Mauremys rivulata* | 41.276186,26.510889 | Türkiye | Edirne | 9.07.2014 | Field study |
| *Mauremys rivulata* | 41.201986,26.913183 | Türkiye | Edirne | 9.07.2014 | Field study |
| *Mauremys rivulata* | 41.168411,26.859053 | Türkiye | Edirne | 9.07.2014 | Field study |
| *Mauremys rivulata* | 41.228617,26.518061 | Türkiye | Edirne | 10.07.2014 | Field study |
| *Mauremys rivulata* | 41.214550,26.562436 | Türkiye | Edirne | 9.07.2014 | Field study |
| *Mauremys rivulata* | 41.196547,26.713386 | Türkiye | Edirne | 8.07.2014 | Field study |
| *Mauremys rivulata* | 41.195333,26.713853 | Türkiye | Edirne | 15.08.2014 | Field study |
| *Mauremys rivulata* | 41.214225,26.476258 | Türkiye | Edirne | 10.07.2014 | Field study |
| *Mauremys rivulata* | 41.212194,26.480883 | Türkiye | Edirne | 10.07.2014 | Field study |
| *Mauremys rivulata* | 41.182206,26.455753 | Türkiye | Edirne | 10.07.2014 | Field study |
| *Mauremys rivulata* | 41.154725,26.456261 | Türkiye | Edirne | 10.07.2014 | Field study |
| *Mauremys rivulata* | 41.159247,26.797000 | Türkiye | Edirne | 9.07.2014 | Field study |
| *Mauremys rivulata* | 41.172178,26.792711 | Türkiye | Edirne | 9.07.2014 | Field study |
| *Mauremys rivulata* | 41.160264,26.758217 | Türkiye | Edirne | 8.07.2014 | Field study |
| *Mauremys rivulata* | 41.113186,26.591319 | Türkiye | Edirne | 10.07.2014 | Field study |
| *Mauremys rivulata* | 41.112947,26.690150 | Türkiye | Edirne | 10.07.2014 | Field study |
| *Mauremys rivulata* | 41.075486,26.739483 | Türkiye | Edirne | 10.07.2014 | Field study |
| *Mauremys rivulata* | 40.962892,26.675306 | Türkiye | Edirne | 11.07.2014 | Field study |
| *Mauremys rivulata* | 40.934036,26.600575 | Türkiye | Edirne | 11.07.2014 | Field study |
| *Mauremys rivulata* | 40.884908,26.506058 | Türkiye | Edirne | 11.07.2014 | Field study |
| *Mauremys rivulata* | 40.972744,26.396081 | Türkiye | Edirne | 11.07.2014 | Field study |
| *Mauremys rivulata* | 40.935500,26.444506 | Türkiye | Edirne | 11.07.2014 | Field study |
| *Mauremys rivulata* | 40.866131,26.691986 | Türkiye | Edirne | 13.07.2014 | Field study |
| *Mauremys rivulata* | 40.832600,26.313928 | Türkiye | Edirne | 12.07.2014 | Field study |
| *Mauremys rivulata* | 40.785569,26.767897 | Türkiye | Edirne | 13.07.2014 | Field study |
| *Mauremys rivulata* | 40.742525,26.711161 | Türkiye | Edirne | 19.08.2014 | Field study |
| *Mauremys rivulata* | 40.753278,26.170564 | Türkiye | Edirne | 20.06.2014 | Field study |
| *Mauremys rivulata* | 40.725278,26.208464 | Türkiye | Edirne | 20.06.2014 | Field study |
| *Mauremys rivulata* | 40.697742,26.635372 | Türkiye | Edirne | 19.08.2014 | Field study |
| *Mauremys rivulata* | 40.726289,26.209311 | Türkiye | Edirne | 20.06.2014 | Field study |
| *Mauremys rivulata* | 40.717033,26.508397 | Türkiye | Edirne | 20.08.2014 | Field study |
| *Mauremys rivulata* | 40.713653,26.098914 | Türkiye | Edirne | 16.07.2014 | Field study |
| *Mauremys rivulata* | 40.638344,26.445997 | Türkiye | Edirne | 20.08.2014 | Field study |
| *Mauremys rivulata* | 40.684117,26.065892 | Türkiye | Edirne | 19.06.2014 | Field study |
| *Mauremys rivulata* | 40.659606,26.075339 | Türkiye | Edirne | 21.06.2014 | Field study |
| *Mauremys rivulata* | 40.601517,26.111142 | Türkiye | Edirne | 21.06.2014 | Field study |
| *Mauremys rivulata* | 40.631247,26.293697 | Türkiye | Edirne | 21.08.2014 | Field study |
| *Mauremys rivulata* | 40.631247,26.293697 | Türkiye | Edirne | 21.08.2014 | Field study |
| *Mauremys rivulata* | 40.595600,26.264986 | Türkiye | Edirne | 21.08.2014 | Field study |
| *Mauremys rivulata* | 40.603075,26.410297 | Türkiye | Edirne | 21.08.2014 | Field study |
| *Mauremys rivulata* | 40.135676,30.175539 | Türkiye | Bilecik | 28.05.2018 | Field study |
| *Mauremys rivulata* | 40.597678,31.656846 | Türkiye | Bolu | 12.08.2016 | Field study |
| *Mauremys rivulata* | 40.774964,32.010920 | Türkiye | Bolu | 12.08.2016 | Field study |
| *Mauremys rivulata* | 40.767176,32.201004 | Türkiye | Bolu | 15.08.2016 | Field study |
| *Mauremys rivulata* | 40.773310,32.236823 | Türkiye | Bolu | 15.08.2016 | Field study |
| *Mauremys rivulata* | 40.796580,32.274620 | Türkiye | Bolu | 15.08.2016 | Field study |
| *Mauremys rivulata* | 37.1436,35.2474 | Türkiye | Adana | 17.09.2023 | <http://www.turkherptil.org/> |
| *Mauremys rivulata* | 41.6926,27.8928 | Türkiye | Kıyıköy, Kırklareli | 20.05.2023 | http://www.turkherptil.org/ |
| *Mauremys rivulata* | 36.3681,30.3549 | Türkiye | Kumluca, Antalya | 18.07.2022 | http://www.turkherptil.org/ |
| *Mauremys rivulata* | 39.7097,27.5145 | Türkiye | Balya, Balıkesir | 21.07.2022 | <http://www.turkherptil.org/> |
| *Mauremys rivulata* | 37.5282,36.3476 | Türkiye | Andırın, Kahramanmaraş | 9.03.2019 | <http://www.turkherptil.org/> |
| *Mauremys rivulata* | 36.7669,30.4605 | Türkiye | Konyaaltı, Antalya | 8.03.2022 | <http://www.turkherptil.org/> |
| *Mauremys rivulata* | 36.87647,28.58637 | Türkiye | Muğla | 17.01.2022 | <http://www.turkherptil.org/> |
| *Mauremys rivulata* | 37.3812,36.7598 | Türkiye | Türkoğlu, Kahramanmaraş | 12.11.2021 | <http://www.turkherptil.org/> |
| *Mauremys rivulata* | 37.0502,27.7581 | Türkiye | Mazı, Muğla | 4.06.2020 | <http://www.turkherptil.org/> |
| *Mauremys rivulata* | 41.8104,27.8416 | Türkiye | İğneada, Kırklareli | 18.10.2020 | <http://www.turkherptil.org/> |
| *Mauremys rivulata* | 38.0291,28.8015 | Türkiye | Denizli | 17.07.2020 | <http://www.turkherptil.org/> |
| *Mauremys rivulata* | 37.9762,28.9864 | Türkiye | Vali Recep Yazıcıoğlu Dam, Denizli | 23.06.2020 | <http://www.turkherptil.org/> |
| *Mauremys rivulata* | 41.16206,26.49760 | Türkiye | Meriç, Edirne | 14.04.2018 | <http://www.turkherptil.org/> |
| *Mauremys rivulata* | 39.6367,27.7645 | Türkiye | Balıkesir | 30.03.2017 | <http://www.turkherptil.org/> |
| *Mauremys rivulata* | 38.13457,27.05597 | Türkiye | Özdere, İzmir | 2.07.2014 | <http://www.turkherptil.org/> |
| *Mauremys rivulata* | 39.6651,26.3317 | Türkiye | Ayvacık, Çanakkale | 14.05.2016 | <http://www.turkherptil.org/> |
| *Mauremys rivulata* | 40.26402,27.17419 | Türkiye | Biga, Çanakkale | 5.03.2016 | <http://www.turkherptil.org/> |
| *Mauremys rivulata* | 39.1646,26.9157 | Türkiye | Dikili, İzmir | 9.11.2015 | <http://www.turkherptil.org/> |
| *Mauremys rivulata* | 37.8532,29.3824 | Türkiye | Denizli | 10.10.2015 | <http://www.turkherptil.org/> |
| *Mauremys rivulata* | 41.16719,28.99352 | Türkiye | Sarıyer, Istanbul | 14.06.2015 | <http://www.turkherptil.org/> |
| *Mauremys rivulata* | 40.67226,29.48805 | Türkiye | Altınova, Yalova | 12.05.2015 | <http://www.turkherptil.org/> |
| *Mauremys rivulata* | 40.85510,29.55169 | Türkiye | Denizli Lake, Kocaeli | 20.06.2015 | <http://www.turkherptil.org/> |
| *Mauremys rivulata* | 40.35071,26.54509 | Türkiye | Gelibolu, Çanakkale | 10.05.2015 | <http://www.turkherptil.org/> |
| *Mauremys rivulata* | 40.0402,27.6098 | Türkiye | Gönen, Balıkesir | 26.06.2012 | <http://www.turkherptil.org/> |
| *Mauremys rivulata* | 39.8574,27.1338 | Türkiye | Kalkım, Çanakkale | 31.01.2015 | <http://www.turkherptil.org/> |
| *Mauremys rivulata* | 41.12896,29.85157 | Türkiye | Ağva, Istanbul | 9.11.2014 | <http://www.turkherptil.org/> |
| *Mauremys rivulata* | 41.14788,28.46175 | Türkiye | Çatalca, Istanbul | 23.08.2014 | <http://www.turkherptil.org/> |
| *Mauremys rivulata* | 39.8057,26.2937 | Türkiye | Ezine, Çanakkale | 18.08.2012 | <http://www.turkherptil.org/> |
| *Mauremys rivulata* | 40.86382,26.60804 | Türkiye | Keşan, Edirne | 22.06.2014 | <http://www.turkherptil.org/> |
| *Mauremys rivulata* | 41.18812,28.93136 | Türkiye | Sarıyer, Istanbul | 1.06.2014 | <http://www.turkherptil.org/> |
| *Mauremys rivulata* | 36.9371,34.6920 | Türkiye | Tarsus, Mersin | 11.04.2013 | <http://www.turkherptil.org/> |
| *Mauremys rivulata* | 36.3697,33.9291 | Türkiye | Silifke, Mersin | 16.02.2014 | <http://www.turkherptil.org/> |
| *Mauremys rivulata* | 40.10566,28.41279 | Türkiye | Tepecik, Bursa | 3.07.2013 | <http://www.turkherptil.org/> |
| *Mauremys rivulata* | 39.8637,28.1393 | Türkiye | Susurluk, Balıkesir | 9.06.2013 | <http://www.turkherptil.org/> |
| *Mauremys rivulata* | 37.73466,30.49771 | Türkiye | Gölcük, Isparta | 25.05.2013 | <http://www.turkherptil.org/> |
| *Mauremys rivulata* | 41.2957,26.6813 | Türkiye | Uzunköprü, Edirne | 5.04.2013 | <http://www.turkherptil.org/> |
| *Mauremys rivulata* | 37.86667,27.60656 | Türkiye | İncirliova, Aydın | 30.04.2011 | <http://www.turkherptil.org/> |
| *Mauremys rivulata* | 41.75674,32.54323 | Türkiye | Amasra, Bartın | 15.08.2011 | <http://www.turkherptil.org/> |
| *Mauremys rivulata* | 36.17619,36.07707 | Türkiye | Defne, Hatay | 23.11.2007 | <http://www.turkherptil.org/> |
| *Mauremys rivulata* | 36.09896,35.96753 | Türkiye | Samandağ, Hatay | 8.03.2011 | <http://www.turkherptil.org/> |
| *Mauremys rivulata* | 36.098333,36.081167 | Türkiye | Asi River, Hatay | 2.09.2000 | https://www.gbif.org/ |
| *Mauremys rivulata* | 36.098333,36.081167 | Türkiye | Asi River, Hatay | 2.09.2000 | https://www.gbif.org/ |
| *Mauremys rivulata* | 36.098333,36.081167 | Türkiye | Asi River, Hatay | 2.09.2000 | https://www.gbif.org/ |
| *Mauremys rivulata* | 36.098333,36.081167 | Türkiye | Asi River, Hatay | 2.09.2000 | https://www.gbif.org/ |
| *Mauremys rivulata* | 36.098333,36.081167 | Türkiye | Asi River, Hatay | 2.09.2000 | https://www.gbif.org/ |
| *Mauremys rivulata* | 36.365167,33.973167 | Türkiye | Silifke, Mersin | 29.08.2000 | https://www.gbif.org/ |
| *Mauremys rivulata* | 36.365167,33.973167 | Türkiye | Silifke, Mersin | 29.08.2000 | https://www.gbif.org/ |
| *Mauremys rivulata* | 36.365167,33.973167 | Türkiye | Silifke, Mersin | 29.08.2000 | https://www.gbif.org/ |
| *Mauremys rivulata* | 36.365167,33.973167 | Türkiye | Silifke, Mersin | 29.08.2000 | https://www.gbif.org/ |
| *Mauremys rivulata* | 36.365167,33.973167 | Türkiye | Silifke, Mersin | 29.08.2000 | https://www.gbif.org/ |
| *Mauremys rivulata* | 36.365167,33.973167 | Türkiye | Silifke, Mersin | 29.08.2000 | https://www.gbif.org/ |
| *Mauremys rivulata* | 36.365167,33.973167 | Türkiye | Silifke, Mersin | 29.08.2000 | https://www.gbif.org/ |
| *Mauremys rivulata* | 36.365167,33.973167 | Türkiye | Silifke, Mersin | 29.08.2000 | https://www.gbif.org/ |
| *Mauremys rivulata* | 36.081783,32.895233 | Türkiye | Anamur, Mersin | 26.08.2000 | https://www.gbif.org/ |
| *Mauremys rivulata* | 36.081783,32.895233 | Türkiye | Anamur, Mersin | 26.08.2000 | https://www.gbif.org/ |
| *Mauremys rivulata* | 36.081783,32.895233 | Türkiye | Anamur, Mersin | 26.08.2000 | https://www.gbif.org/ |
| *Mauremys rivulata* | 37.043121,28.329569 | Türkiye | Akyaka, Muğla | 7.08.2015 | https://www.gbif.org/ |
| *Mauremys rivulata* | 37.043121,28.329569 | Türkiye | Akyaka, Muğla | 5.08.2015 | https://www.gbif.org/ |
| *Mauremys rivulata* | 37.042984,28.343937 | Türkiye | Akyaka, Muğla | 11.08.2015 | https://www.gbif.org/ |
| *Mauremys rivulata* | 37.042984,28.343937 | Türkiye | Akyaka, Muğla | 9.08.2015 | https://www.gbif.org/ |
| *Mauremys rivulata* | 37.043121,28.329569 | Türkiye | Akyaka, Muğla | 13.08.2015 | https://www.gbif.org/ |
| *Mauremys rivulata* | 40.18333,28.89054 | Türkiye | Uludağ, Bursa | 7.03.1999 | https://www.gbif.org/ |
| *Mauremys rivulata* | 36.033817,32.81275 | Türkiye | Anamur, Mersin | 23.08.2000 | https://www.gbif.org/ |
| *Mauremys rivulata* | 36.033817,32.81275 | Türkiye | Anamur, Mersin | 23.08.2000 | https://www.gbif.org/ |
| *Mauremys rivulata* | 36.778629,34.860477 | Türkiye | Tarsus, Mersin | 25.12.2006 | https://www.gbif.org/ |
| *Mauremys rivulata* | 36.08091,32.894524 | Türkiye | Anamur, Mersin | 10.4.2010 | https://www.gbif.org/ |
| *Mauremys rivulata* | 36.301846,33.947411 | Türkiye | Göksu | 25.3.2011 | https://www.gbif.org/ |
| *Mauremys rivulata* | 36.336422,34.027748 | Türkiye | Göksu | 24.3.2011 | https://www.gbif.org/ |
| *Mauremys rivulata* | 37.228416,27.684174 | Türkiye | Milas, Muğla | 24.10.1989 | https://www.gbif.org/ |
| *Mauremys rivulata* | 36.301846,33.947411 | Türkiye | Göksu | 29.3.2011 | https://www.gbif.org/ |
| *Mauremys rivulata* | 36.998703,27.651037 | Türkiye | Muğla | 29.6.2024 | https://www.gbif.org/ |
| *Mauremys rivulata* | 37.439017,27.412587 | Türkiye | Muğla | 30.6.2024 | https://www.gbif.org/ |
| *Mauremys rivulata* | 36.82,28.62 | Türkiye | Dalyan, Muğla | 4.3.2024 | https://www.gbif.org/ |
| *Mauremys rivulata* | 36.843217,28.633578 | Türkiye | Dalyan, Muğla | 22.5.2024 | https://www.gbif.org/ |
| *Mauremys rivulata* | 36.8226,28.6386 | Türkiye | Dalyan, Muğla | 5.8.2015 | https://www.gbif.org/ |
| *Mauremys rivulata* | 36.397343,30.474203 | Türkiye | Antalya | 4.4.2024 | https://www.gbif.org/ |
| *Mauremys rivulata* | 36.825211,28.619074 | Türkiye | Dalyan, Muğla | 6.4.2024 | https://www.gbif.org/ |
| *Mauremys rivulata* | 36.810345,28.641632 | Türkiye | Dalyan, Muğla | 6.4.2024 | https://www.gbif.org/ |
| *Mauremys rivulata* | 36.39,30.47 | Türkiye | Antalya | 4.4.2024 | https://www.gbif.org/ |
| *Mauremys rivulata* | 36.82,28.62 | Türkiye | Dalyan, Muğla | 6.4.2024 | https://www.gbif.org/ |
| *Mauremys rivulata* | 36.830763,28.699792 | Türkiye | Dalyan, Muğla | 19.2.2013 | https://www.gbif.org/ |
| *Mauremys rivulata* | 36.816073,28.640628 | Türkiye | Dalyan, Muğla | 8.7.2009 | https://www.gbif.org/ |
| *Mauremys rivulata* | 36.8226,28.6386 | Türkiye | Dalyan, Muğla | 5.8.2015 | https://www.gbif.org/ |
| *Mauremys rivulata* | 36.8203,28.64007 | Türkiye | Dalyan, Muğla | 10.7.2009 | https://www.gbif.org/ |
| *Mauremys rivulata* | 36.39,30.47 | Türkiye | Antalya | 29.2.2024 | https://www.gbif.org/ |
| *Mauremys rivulata* | 37.828884,27.791523 | Türkiye | Aydın | 3.5.2024 | https://www.gbif.org/ |
| *Mauremys rivulata* | 37.002778,30.820147 | Türkiye | Antalya | 9.5.2024 | https://www.gbif.org/ |
| *Mauremys rivulata* | 36.765799,31.409517 | Türkiye | Antalya | 8.5.2024 | https://www.gbif.org/ |
| *Mauremys rivulata* | 36.879337,30.656939 | Türkiye | Antalya | 15.4.2024 | https://www.gbif.org/ |
| *Mauremys rivulata* | 36.230279,29.957503 | Türkiye | Antalya | 18.12.2022 | https://www.gbif.org/ |
| *Mauremys rivulata* | 36.879325,30.656922 | Türkiye | Antalya | 30.3.2024 | https://www.gbif.org/ |
| *Mauremys rivulata* | 41.20783,29.233036 | Türkiye | Istanbul | 1.5.2014 | https://www.gbif.org/ |
| *Mauremys rivulata* | 37.134062,36.227687 | Türkiye | Osmaniye | 5.6.2013 | https://www.gbif.org/ |
| *Mauremys rivulata* | 40.392712,28.7992 | Türkiye | Bursa | 14.3.2024 | https://www.gbif.org/ |
| *Mauremys rivulata* | 40.392712,28.7992 | Türkiye | Bursa | 14.3.2024 | https://www.gbif.org/ |
| *Mauremys rivulata* | 36.968538,27.285432 | Türkiye | Muğla | 16.10.2017 | https://www.gbif.org/ |
| *Mauremys rivulata* | 36.968963,27.284926 | Türkiye | Muğla | 20.10.2019 | https://www.gbif.org/ |
| *Mauremys rivulata* | 39.631852,26.964145 | Türkiye | Balikesir | 8.10.2023 | https://www.gbif.org/ |
| *Mauremys rivulata* | 40.256658,28.53713 | Türkiye | Bursa | 7.5.2018 | https://www.gbif.org/ |
| *Mauremys rivulata* | 36.684074,29.0907 | Türkiye | Muğla | 15.4.1995 | https://www.gbif.org/ |
| *Mauremys rivulata* | 36.877878,30.657079 | Türkiye | Antalya | 13.12.2023 | https://www.gbif.org/ |
| *Mauremys rivulata* | 37.941066,27.342692 | Türkiye | Izmir | 17.9.2017 | https://www.gbif.org/ |
| *Mauremys rivulata* | 36.82,28.62 | Türkiye | Dalyan | 1.11.2023 | https://www.gbif.org/ |
| *Mauremys rivulata* | 36.69,28.78 | Türkiye | Muğla | 19.10.2023 | https://www.gbif.org/ |
| *Mauremys rivulata* | 36.862046,30.907218 | Türkiye | Antalya | 24.5.2023 | https://www.gbif.org/ |
| *Mauremys rivulata* | 36.840846,28.633917 | Türkiye | Dalyan, Muğla | 1.7.2008 | https://www.gbif.org/ |
| *Mauremys rivulata* | 36.82,28.66 | Türkiye | Dalyan, Muğla | 29.10.2023 | https://www.gbif.org/ |
| *Mauremys rivulata* | 36.396423,30.475676 | Türkiye | Antalya | 6.8.2023 | https://www.gbif.org/ |
| *Mauremys rivulata* | 36.743721,31.486958 | Türkiye | Manavgat, Antalya | 28.10.2021 | https://www.gbif.org/ |
| *Mauremys rivulata* | 36.802579,31.352327 | Türkiye | Side, Antalya | 13.10.2023 | https://www.gbif.org/ |
| *Mauremys rivulata* | 36.82,28.62 | Türkiye | Dalyan, Muğla | 28.10.2023 | https://www.gbif.org/ |
| *Mauremys rivulata* | 36.26,29.31 | Türkiye | Antalya | 24.10.2023 | https://www.gbif.org/ |
| *Mauremys rivulata* | 36.862353,30.907649 | Türkiye | Antalya | 24.5.2023 | https://www.gbif.org/ |
| *Mauremys rivulata* | 36.829956,28.642708 | Türkiye | Dalyan, Muğla | 10.6.2022 | https://www.gbif.org/ |
| *Mauremys rivulata* | 37.856023,29.38586 | Türkiye | Denizli | 7.8.2023 | https://www.gbif.org/ |
| *Mauremys rivulata* | 36.825653,28.625058 | Türkiye | Dalyan, Muğla | 22.4.2005 | https://www.gbif.org/ |
| *Mauremys rivulata* | 36.814793,28.635938 | Türkiye | Dalyan, Muğla | 27.5.2019 | https://www.gbif.org/ |
| *Mauremys rivulata* | 36.83,28.68 | Türkiye | Dalyan, Muğla | 29.10.2023 | https://www.gbif.org/ |
| *Mauremys rivulata* | 36.396484,30.475587 | Türkiye | Antalya | 6.8.2023 | https://www.gbif.org/ |
| *Mauremys rivulata* | 36.862042,30.907314 | Türkiye | Antalya | 24.5.2023 | https://www.gbif.org/ |
| *Mauremys rivulata* | 36.625901,31.769835 | Türkiye | Antalya | 29.11.2022 | https://www.gbif.org/ |
| *Mauremys rivulata* | 36.737633,31.494572 | Türkiye | Manavgat, Antalya | 28.10.2021 | https://www.gbif.org/ |
| *Mauremys rivulata* | 36.740551,31.493811 | Türkiye | Manavgat, Antalya | 28.10.2021 | https://www.gbif.org/ |
| *Mauremys rivulata* | 36.87471,31.546772 | Türkiye | Manavgat, Antalya | 31.10.2021 | https://www.gbif.org/ |
| *Mauremys rivulata* | 37.93744,27.344827 | Türkiye | Izmir | 16.5.2012 | https://www.gbif.org/ |
| *Mauremys rivulata* | 36.618772,35.499083 | Türkiye | Adana | 27.9.2006 | https://www.gbif.org/ |
| *Mauremys rivulata* | 39.583978,26.110337 | Türkiye | Çanakkale | 29.7.2006 | https://www.gbif.org/ |
| *Mauremys rivulata* | 36.967623,28.693537 | Türkiye | Muğla | 14.10.2023 | https://www.gbif.org/ |
| *Mauremys rivulata* | 39.118992,27.164947 | Türkiye | Izmir | 14.10.2023 | https://www.gbif.org/ |
| *Mauremys rivulata* | 39.118972,27.164961 | Türkiye | Izmir | 14.10.2023 | https://www.gbif.org/ |
| *Mauremys rivulata* | 36.879631,30.65744 | Türkiye | Antalya | 21.9.2023 | https://www.gbif.org/ |
| *Mauremys rivulata* | 38.105301,28.21273 | Türkiye | Izmir | 26.8.2023 | https://www.gbif.org/ |
| *Mauremys rivulata* | 36.877945,30.656966 | Türkiye | Antalya | 19.9.2023 | https://www.gbif.org/ |
| *Mauremys rivulata* | 36.873944,30.620292 | Türkiye | Antalya | 31.8.2023 | https://www.gbif.org/ |
| *Mauremys rivulata* | 36.754082,31.688428 | Türkiye | Antalya | 16.5.2016 | https://www.gbif.org/ |
| *Mauremys rivulata* | 36.874394,30.620067 | Türkiye | Antalya | 24.9.2023 | https://www.gbif.org/ |
| *Mauremys rivulata* | 36.741287,30.565056 | Türkiye | Antalya | 11.9.2023 | https://www.gbif.org/ |
| *Mauremys rivulata* | 36.839109,28.632977 | Türkiye | Dalyan, Muğla | 28.5.2009 | https://www.gbif.org/ |
| *Mauremys rivulata* | 36.913475,28.692202 | Türkiye | Dalyan, Muğla | 1.6.2009 | https://www.gbif.org/ |
| *Mauremys rivulata* | 37.934961,27.256242 | Türkiye | Izmir | 10.10.2009 | https://www.gbif.org/ |
| *Mauremys rivulata* | 38.069103,26.918568 | Türkiye | Izmir | 21.8.2023 | https://www.gbif.org/ |
| *Mauremys rivulata* | 40.511031,28.825048 | Türkiye | Yalova | 10.8.2023 | https://www.gbif.org/ |
| *Mauremys rivulata* | 36.877765,30.657183 | Türkiye | Antalya | 13.8.2023 | https://www.gbif.org/ |
| *Mauremys rivulata* | 40.519971,28.828919 | Türkiye | Yalova | 9.8.2023 | https://www.gbif.org/ |
| *Mauremys rivulata* | 40.510916,28.824849 | Türkiye | Yalova | 10.8.2023 | https://www.gbif.org/ |
| *Mauremys rivulata* | 36.960685,28.701983 | Türkiye | Muğla | 13.6.2016 | https://www.gbif.org/ |
| *Mauremys rivulata* | 36.08091,32.894524 | Türkiye | Anamur, Mersin | 21.3.2008 | https://www.gbif.org/ |
| *Mauremys rivulata* | 36.605442,30.563628 | Türkiye | Antalya | 27.7.2023 | https://www.gbif.org/ |
| *Mauremys rivulata* | 36.803753,31.351901 | Türkiye | Antalya | 1.3.2016 | https://www.gbif.org/ |
| *Mauremys rivulata* | 36.80685,31.350389 | Türkiye | Antalya | 2.3.2016 | https://www.gbif.org/ |
| *Mauremys rivulata* | 36.805073,31.3523 | Türkiye | Antalya | 1.3.2016 | https://www.gbif.org/ |
| *Mauremys rivulata* | 36.804103,31.352019 | Türkiye | Antalya | 1.3.2016 | https://www.gbif.org/ |
| *Mauremys rivulata* | 36.804103,31.352019 | Türkiye | Antalya | 1.3.2016 | https://www.gbif.org/ |
| *Mauremys rivulata* | 36.849406,28.284067 | Türkiye | Muğla | 27.6.2023 | https://www.gbif.org/ |
| *Mauremys rivulata* | 36.68311,30.52872 | Türkiye | Antalya | 17.8.2022 | https://www.gbif.org/ |
| *Mauremys rivulata* | 36.400547,30.458012 | Türkiye | Antalya | 4.7.2023 | https://www.gbif.org/ |
| *Mauremys rivulata* | 36.321964,33.903068 | Türkiye | Mersin | 7.10.2010 | https://www.gbif.org/ |
| *Mauremys rivulata* | 40.318506,28.510437 | Türkiye | Bursa | 21.6.2023 | https://www.gbif.org/ |
| *Mauremys rivulata* | 36.403866,30.472918 | Türkiye | Antalya | 20.6.2023 | https://www.gbif.org/ |
| *Mauremys rivulata* | 36.810145,31.342463 | Türkiye | Antalya | 9.6.2023 | https://www.gbif.org/ |
| *Mauremys rivulata* | 36.722908,28.753945 | Türkiye | Muğla | 29.5.2023 | https://www.gbif.org/ |
| *Mauremys rivulata* | 36.68256,30.528104 | Türkiye | Antalya | 2.5.2023 | https://www.gbif.org/ |
| *Mauremys rivulata* | 41.015491,29.047806 | Türkiye | Istanbul | 4.6.2023 | https://www.gbif.org/ |
| *Mauremys rivulata* | 41.015724,29.047948 | Türkiye | Istanbul | 4.6.2023 | https://www.gbif.org/ |
| *Mauremys rivulata* | 40.999669,28.880109 | Türkiye | Istanbul | 15.5.2023 | https://www.gbif.org/ |
| *Mauremys rivulata* | 40.65821,29.266608 | Türkiye | Yalova | 7.7.2021 | https://www.gbif.org/ |
| *Mauremys rivulata* | 36.077286,32.832889 | Türkiye | Mersin | 11.7.2010 | https://www.gbif.org/ |
| *Mauremys rivulata* | 41.103425,29.029742 | Türkiye | Istanbul | 22.5.2023 | https://www.gbif.org/ |
| *Mauremys rivulata* | 41.391699,28.4523 | Türkiye | Istanbul | 22.4.2023 | https://www.gbif.org/ |
| *Mauremys rivulata* | 36.835638,30.592405 | Türkiye | Antalya | 1.3.2020 | https://www.gbif.org/ |
| *Mauremys rivulata* | 36.87929,30.65689 | Türkiye | Antalya | 3.5.2023 | https://www.gbif.org/ |
| *Mauremys rivulata* | 36.687888,28.784205 | Türkiye | Muğla | 24.4.2023 | https://www.gbif.org/ |
| *Mauremys rivulata* | 36.882728,30.664108 | Türkiye | Antalya | 22.4.2023 | https://www.gbif.org/ |
| *Mauremys rivulata* | 36.827329,28.655418 | Türkiye | Muğla | 11.4.2023 | https://www.gbif.org/ |
| *Mauremys rivulata* | 36.297659,33.924746 | Türkiye | Mersin | 2.4.2023 | https://www.gbif.org/ |
| *Mauremys rivulata* | 36.84507,30.589062 | Türkiye | Antalya | 15.3.2023 | https://www.gbif.org/ |
| *Mauremys rivulata* | 41.133051,29.032238 | Türkiye | Istanbul | 26.2.2023 | https://www.gbif.org/ |
| *Mauremys rivulata* | 39.11885,27.164961 | Türkiye | Izmir | 24.2.2023 | https://www.gbif.org/ |
| *Mauremys rivulata* | 36.968725,28.271903 | Türkiye | Muğla | 7.6.2019 | https://www.gbif.org/ |
| *Mauremys rivulata* | 36.823982,28.760229 | Türkiye | Muğla | 8.6.2013 | https://www.gbif.org/ |
| *Mauremys rivulata* | 36.809375,31.342127 | Türkiye | Antalya | 12.4.2022 | https://www.gbif.org/ |
| *Mauremys rivulata* | 36.854629,30.807707 | Türkiye | Antalya | 10.4.2022 | https://www.gbif.org/ |
| *Mauremys rivulata* | 36.681787,30.527964 | Türkiye | Antalya | 4.8.2017 | https://www.gbif.org/ |
| *Mauremys rivulata* | 37.164801,36.13997 | Türkiye | Osmaniye | 3.5.2014 | https://www.gbif.org/ |
| *Mauremys rivulata* | 36.825439,28.619935 | Türkiye | Muğla | 20.12.2022 | https://www.gbif.org/ |
| *Mauremys rivulata* | 36.403793,30.473044 | Türkiye | Antalya | 23.5.2022 | https://www.gbif.org/ |
| *Mauremys rivulata* | 36.664928,34.410972 | Türkiye | Mersin | 15.11.2022 | https://www.gbif.org/ |
| *Mauremys rivulata* | 36.401663,30.474723 | Türkiye | Antalya | 22.5.2022 | https://www.gbif.org/ |
| *Mauremys rivulata* | 36.229962,29.996653 | Türkiye | Antalya | 22.10.2022 | https://www.gbif.org/ |
| *Mauremys rivulata* | 36.230158,29.99803 | Türkiye | Antalya | 22.10.2022 | https://www.gbif.org/ |
| *Mauremys rivulata* | 36.332238,33.950795 | Türkiye | Mersin | 24.3.1993 | https://www.gbif.org/ |
| *Mauremys rivulata* | 36.414189,30.477703 | Türkiye | Antalya | 12.10.2022 | https://www.gbif.org/ |
| *Mauremys rivulata* | 36.319856,34.034513 | Türkiye | Mersin | 26.4.1991 | https://www.gbif.org/ |
| *Mauremys rivulata* | 36.663893,29.114271 | Türkiye | Muğla | 2.10.2022 | https://www.gbif.org/ |
| *Mauremys rivulata* | 36.639491,31.758229 | Türkiye | Antalya | 2.1.2018 | https://www.gbif.org/ |
| *Mauremys rivulata* | 36.814822,28.63587 | Türkiye | Muğla | 1.10.2022 | https://www.gbif.org/ |
| *Mauremys rivulata* | 36.306927,33.966014 | Türkiye | Mersin | 11.4.1991 | https://www.gbif.org/ |
| *Mauremys rivulata* | 39.63067,27.41693 | Türkiye | Balikesir | 31.8.2022 | https://www.gbif.org/ |
| *Mauremys rivulata* | 39.630117,27.416333 | Türkiye | Balikesir | 31.8.2022 | https://www.gbif.org/ |
| *Mauremys rivulata* | 37.114527,27.398597 | Türkiye | Muğla | 31.8.2022 | https://www.gbif.org/ |
| *Mauremys rivulata* | 36.905626,28.401857 | Türkiye | Muğla | 23.8.2022 | https://www.gbif.org/ |
| *Mauremys rivulata* | 36.817446,31.31523 | Türkiye | Antalya | 18.7.2012 | https://www.gbif.org/ |
| *Mauremys rivulata* | 36.401628,30.474987 | Türkiye | Antalya | 19.7.2022 | https://www.gbif.org/ |
| *Mauremys rivulata* | 36.930662,30.571084 | Türkiye | Antalya | 20.5.2022 | https://www.gbif.org/ |
| *Mauremys rivulata* | 37.002818,30.821125 | Türkiye | Antalya | 11.5.2022 | https://www.gbif.org/ |
| *Mauremys rivulata* | 36.820249,28.626864 | Türkiye | Muğla | 13.7.2018 | https://www.gbif.org/ |
| *Mauremys rivulata* | 36.081086,32.89447 | Türkiye | Mersin | 14.7.1989 | https://www.gbif.org/ |
| *Mauremys rivulata* | 36.830845,28.64234 | Türkiye | Muğla | 10.6.2022 | https://www.gbif.org/ |
| *Mauremys rivulata* | 36.081661,32.895359 | Türkiye | Mersin | 10.6.2022 | https://www.gbif.org/ |
| *Mauremys rivulata* | 41.17688,28.984873 | Türkiye | Istanbul | 22.6.2022 | https://www.gbif.org/ |
| *Mauremys rivulata* | 39.17464,27.153071 | Türkiye | Izmir | 3.7.1989 | https://www.gbif.org/ |
| *Mauremys rivulata* | 36.859603,30.729988 | Türkiye | Antalya | 9.6.2022 | https://www.gbif.org/ |
| *Mauremys rivulata* | 36.321436,30.184271 | Türkiye | Antalya | 17.12.2009 | https://www.gbif.org/ |
| *Mauremys rivulata* | 36.340122,30.190215 | Türkiye | Antalya | 6.12.2009 | https://www.gbif.org/ |
| *Mauremys rivulata* | 40.028792,32.946935 | Türkiye | Ankara | 22.5.2022 | https://www.gbif.org/ |
| *Mauremys rivulata* | 36.797941,28.648735 | Türkiye | Muğla | 11.5.2022 | https://www.gbif.org/ |
| *Mauremys rivulata* | 36.957424,28.688658 | Türkiye | Muğla | 11.5.2022 | https://www.gbif.org/ |
| *Mauremys rivulata* | 36.825356,28.616753 | Türkiye | Muğla | 12.5.2022 | https://www.gbif.org/ |
| *Mauremys rivulata* | 36.840176,28.62491 | Türkiye | Muğla | 12.5.2022 | https://www.gbif.org/ |
| *Mauremys rivulata* | 36.845093,28.628199 | Türkiye | Muğla | 12.5.2022 | https://www.gbif.org/ |
| *Mauremys rivulata* | 36.825203,28.623989 | Türkiye | Muğla | 13.5.2022 | https://www.gbif.org/ |
| *Mauremys rivulata* | 36.845093,28.628199 | Türkiye | Muğla | 12.5.2022 | https://www.gbif.org/ |
| *Mauremys rivulata* | 36.836891,28.614849 | Türkiye | Muğla | 13.5.2022 | https://www.gbif.org/ |
| *Mauremys rivulata* | 36.306849,29.315668 | Türkiye | Antalya | 7.4.2022 | https://www.gbif.org/ |
| *Mauremys rivulata* | 36.315242,33.903564 | Türkiye | Mersin | 9.5.2022 | https://www.gbif.org/ |
| *Mauremys rivulata* | 36.776813,28.633967 | Türkiye | Muğla | 11.5.2022 | https://www.gbif.org/ |
| *Mauremys rivulata* | 40.99187,28.7315 | Türkiye | Istanbul | 16.5.2016 | https://www.gbif.org/ |
| *Mauremys rivulata* | 36.422749,30.482887 | Türkiye | Antalya | 28.4.2022 | https://www.gbif.org/ |
| *Mauremys rivulata* | 36.810614,31.341951 | Türkiye | Antalya | 12.4.2022 | https://www.gbif.org/ |
| *Mauremys rivulata* | 36.858618,30.860087 | Türkiye | Antalya | 10.4.2022 | https://www.gbif.org/ |
| *Mauremys rivulata* | 36.84963,30.808246 | Türkiye | Antalya | 10.4.2022 | https://www.gbif.org/ |
| *Mauremys rivulata* | 36.234322,29.967522 | Türkiye | Antalya | 10.4.2022 | https://www.gbif.org/ |
| *Mauremys rivulata* | 37.949704,27.363913 | Türkiye | Izmir | 22.4.2016 | https://www.gbif.org/ |
| *Mauremys rivulata* | 37.949665,27.363881 | Türkiye | Izmir | 11.8.2014 | https://www.gbif.org/ |
| *Mauremys rivulata* | 36.846898,28.286483 | Türkiye | Muğla | 15.3.2022 | https://www.gbif.org/ |
| *Mauremys rivulata* | 36.305005,33.936682 | Türkiye | Mersin | 16.3.2022 | https://www.gbif.org/ |
| *Mauremys rivulata* | 36.909219,28.598082 | Türkiye | Dalyan, Muğla | 28.2.2020 | https://www.gbif.org/ |
| *Mauremys rivulata* | 36.909126,28.598072 | Türkiye | Dalyan, Muğla | 28.2.2020 | https://www.gbif.org/ |
| *Mauremys rivulata* | 36.939648,28.645941 | Türkiye | Dalyan, Muğla | 25.2.2020 | https://www.gbif.org/ |
| *Mauremys rivulata* | 36.907997,28.595806 | Türkiye | Dalyan, Muğla | 28.2.2020 | https://www.gbif.org/ |
| *Mauremys rivulata* | 36.939589,28.645957 | Türkiye | Dalyan, Muğla | 25.2.2020 | https://www.gbif.org/ |
| *Mauremys rivulata* | 36.958926,28.657279 | Türkiye | Dalyan, Muğla | 25.2.2020 | https://www.gbif.org/ |
| *Mauremys rivulata* | 36.958904,28.657309 | Türkiye | Dalyan, Muğla | 25.2.2020 | https://www.gbif.org/ |
| *Mauremys rivulata* | 36.309012,30.464724 | Türkiye | Antalya | 11.9.2020 | https://www.gbif.org/ |
| *Mauremys rivulata* | 36.834389,30.583012 | Türkiye | Antalya | 8.3.2022 | https://www.gbif.org/ |
| *Mauremys rivulata* | 38.566643,26.892239 | Türkiye | Izmir | 16.5.2019 | https://www.gbif.org/ |
| *Mauremys rivulata* | 36.650732,35.353554 | Türkiye | Adana | 16.5.2011 | https://www.gbif.org/ |
| *Mauremys rivulata* | 36.819399,28.630469 | Türkiye | Muğla | 17.1.2022 | https://www.gbif.org/ |
| *Mauremys rivulata* | 36.859894,30.729978 | Türkiye | Antalya | 2.11.2021 | https://www.gbif.org/ |
| *Mauremys rivulata* | 37.773712,29.117823 | Türkiye | Denizli | 14.7.2019 | https://www.gbif.org/ |
| *Mauremys rivulata* | 36.853745,30.782013 | Türkiye | Antalya | 20.11.2016 | https://www.gbif.org/ |
| *Mauremys rivulata* | 38.442904,35.504154 | Türkiye | Kayseri | 10.10.2021 | https://www.gbif.org/ |
| *Mauremys rivulata* | 40.415378,26.723613 | Türkiye | Çanakkale | 31.7.2021 | https://www.gbif.org/ |
| *Mauremys rivulata* | 36.854272,28.606674 | Türkiye | Muğla | 18.10.2021 | https://www.gbif.org/ |
| *Mauremys rivulata* | 36.754677,31.47361 | Türkiye | Manavgat, Antalya | 30.5.2019 | https://www.gbif.org/ |
| *Mauremys rivulata* | 39.993306,32.650591 | Türkiye | Ankara | 25.7.2020 | https://www.gbif.org/ |
| *Mauremys rivulata* | 36.760033,31.410331 | Türkiye | Antalya | 3.5.2021 | https://www.gbif.org/ |
| *Mauremys rivulata* | 39.319172,26.692387 | Türkiye | Balikesir | 8.9.2021 | https://www.gbif.org/ |
| *Mauremys rivulata* | 38.059006,27.016325 | Türkiye | Izmir | 20.8.2021 | https://www.gbif.org/ |
| *Mauremys rivulata* | 36.59708,31.990898 | Türkiye | Antalya | 15.3.2016 | https://www.gbif.org/ |
| *Mauremys rivulata* | 41.014489,29.047868 | Türkiye | Istanbul | 22.6.2021 | https://www.gbif.org/ |
| *Mauremys rivulata* | 40.995967,28.728753 | Türkiye | Istanbul | 7.6.2021 | https://www.gbif.org/ |
| *Mauremys rivulata* | 36.759987,31.41033 | Türkiye | Antalya | 3.5.2021 | https://www.gbif.org/ |
| *Mauremys rivulata* | 36.746343,31.490143 | Türkiye | Antalya | 28.4.2021 | https://www.gbif.org/ |
| *Mauremys rivulata* | 36.969299,28.668817 | Türkiye | Muğla | 28.4.2021 | https://www.gbif.org/ |
| *Mauremys rivulata* | 36.870885,30.717815 | Türkiye | Antalya | 12.5.2019 | https://www.gbif.org/ |
| *Mauremys rivulata* | 36.846565,34.783772 | Türkiye | Mersin | 19.4.2019 | https://www.gbif.org/ |
| *Mauremys rivulata* | 36.871777,28.645793 | Türkiye | Muğla | 30.3.2014 | https://www.gbif.org/ |
| *Mauremys rivulata* | 36.395757,30.473484 | Türkiye | Antalya | 15.5.2015 | https://www.gbif.org/ |
| *Mauremys rivulata* | 37.249235,27.573165 | Türkiye | Muğla | 12.5.2019 | https://www.gbif.org/ |
| *Mauremys rivulata* | 36.806952,31.350434 | Türkiye | Antalya | 2.3.2016 | https://www.gbif.org/ |
| *Mauremys rivulata* | 36.270945,29.316814 | Türkiye | Antalya | 9.4.2012 | https://www.gbif.org/ |
| *Mauremys rivulata* | 36.851256,28.463815 | Türkiye | Muğla | 28.4.2020 | https://www.gbif.org/ |
| *Mauremys rivulata* | 36.39485,30.470947 | Türkiye | Antalya | 19.9.2020 | https://www.gbif.org/ |
| *Mauremys rivulata* | 36.862753,28.631435 | Türkiye | Muğla | 24.10.2008 | https://www.gbif.org/ |
| *Mauremys rivulata* | 36.83,31.28 | Türkiye | Antalya | 4.9.2014 | https://www.gbif.org/ |
| *Mauremys rivulata* | 36.8,28.65 | Türkiye | Muğla | 5.5.2014 | https://www.gbif.org/ |
| *Mauremys rivulata* | 36.79,28.63 | Türkiye | Muğla | 4.5.2014 | https://www.gbif.org/ |
| *Mauremys rivulata* | 36.83,28.63 | Türkiye | Muğla | 6.5.2014 | https://www.gbif.org/ |
| *Mauremys rivulata* | 36.83,31.28 | Türkiye | Antalya | 1.8.2015 | https://www.gbif.org/ |
| *Mauremys rivulata* | 37.949579,27.363913 | Türkiye | Izmir | 5.8.2019 | https://www.gbif.org/ |
| *Mauremys rivulata* | 38.989774,35.243322 | Türkiye | Kayseri | 31.7.2020 | https://www.gbif.org/ |
| *Mauremys rivulata* | 38.277108,26.378713 | Türkiye | Izmir | 5.1.2020 | https://www.gbif.org/ |
| *Mauremys rivulata* | 38.277108,26.378713 | Türkiye | Izmir | 5.1.2020 | https://www.gbif.org/ |
| *Mauremys rivulata* | 36.3489,30.208433 | Türkiye | Antalya | 7.10.2020 | https://www.gbif.org/ |
| *Mauremys rivulata* | 36.341905,30.169658 | Türkiye | Antalya | 7.10.2020 | https://www.gbif.org/ |
| *Mauremys rivulata* | 36.08164,32.895468 | Türkiye | Mersin | 4.5.2015 | https://www.gbif.org/ |
| *Mauremys rivulata* | 36.08161,32.895517 | Türkiye | Mersin | 4.5.2015 | https://www.gbif.org/ |
| *Mauremys rivulata* | 36.959103,28.657078 | Türkiye | Dalyan, Muğla | 25.2.2020 | https://www.gbif.org/ |
| *Mauremys rivulata* | 36.959167,28.657499 | Türkiye | Dalyan, Muğla | 25.2.2020 | https://www.gbif.org/ |
| *Mauremys rivulata* | 36.826233,28.659707 | Türkiye | Dalyan, Muğla | 22.4.2014 | https://www.gbif.org/ |
| *Mauremys rivulata* | 36.809695,28.6191 | Türkiye | Dalyan, Muğla | 5.8.2015 | https://www.gbif.org/ |
| *Mauremys rivulata* | 36.339,33.9443 | Türkiye | Göksu Delta | 4.5.2015 | https://www.gbif.org/ |
| *Mauremys rivulata* | 37.195283,36.081245 | Türkiye | Adana | 17.6.2014 | https://www.gbif.org/ |
| *Mauremys rivulata* | 36.742541,31.492631 | Türkiye | Manavgat, Antalya | 4.7.2015 | https://www.gbif.org/ |
| *Mauremys rivulata* | 36.851272,28.661419 | Türkiye | Dalyan, Muğla | 26.4.2014 | https://www.gbif.org/ |
| *Mauremys rivulata* | 36.822878,28.646091 | Türkiye | Dalyan, Muğla | 2.5.2014 | https://www.gbif.org/ |
| *Mauremys rivulata* | 36.815711,31.304172 | Türkiye | Side, Antalya | 24.4.2005 | https://www.gbif.org/ |
| *Mauremys rivulata* | 36.725439,31.571452 | Türkiye | Antalya | 27.4.2005 | https://www.gbif.org/ |
| *Mauremys rivulata* | 36.272234,29.309807 | Türkiye | Antalya | 9.3.2014 | https://www.gbif.org/ |
| *Mauremys rivulata* | 36.819248,28.655176 | Türkiye | Dalyan, Muğla | 15.10.2013 | https://www.gbif.org/ |
| *Mauremys rivulata* | 36.739142,31.493106 | Türkiye | Manavgat, Antalya | 27.5.2018 | https://www.gbif.org/ |
| *Mauremys rivulata* | 36.935349,31.173134 | Türkiye | Aspendos, Antalya | 26.12.2014 | https://www.gbif.org/ |
| *Mauremys rivulata* | 36.745883,35.266353 | Türkiye | Adana | 25.9.2011 | https://www.gbif.org/ |
| *Mauremys rivulata* | 36.839216,28.636547 | Türkiye | Dalyan, Muğla | 9.6.2013 | https://www.gbif.org/ |
| *Mauremys rivulata* | 36.84343,28.631916 | Türkiye | Dalyan, Muğla | 23.5.2013 | https://www.gbif.org/ |
| *Mauremys rivulata* | 36.837729,28.716202 | Türkiye | Dalyan, Muğla | 16.10.2013 | https://www.gbif.org/ |
| *Mauremys rivulata* | 36.78,28.62 | Türkiye | Dalyan, Muğla | 14.10.2013 | https://www.gbif.org/ |
| *Mauremys rivulata* | 37.177462,27.606725 | Türkiye | Muğla | 10.8.2011 | https://www.gbif.org/ |
| *Mauremys rivulata* | 36.321803,33.963301 | Türkiye | Mersin | 13.4.1993 | https://www.gbif.org/ |
| *Mauremys rivulata* | 36.9,28.76 | Türkiye | Dalyan, Muğla | 3.5.2014 | https://www.gbif.org/ |
| *Mauremys rivulata* | 36.744119,31.486359 | Türkiye | Manavgat, Antalya | 11.5.2013 | https://www.gbif.org/ |
| *Mauremys rivulata* | 36.312832,30.462831 | Türkiye | Antalya | 20.5.2013 | https://www.gbif.org/ |
| *Mauremys rivulata* | 36.748711,28.878511 | Türkiye | Muğla | 24.5.2015 | https://www.gbif.org/ |
| *Mauremys rivulata* | 36.2936,29.2619 | Türkiye | Muğla | 29.10.2015 | https://www.gbif.org/ |
| *Mauremys rivulata* | 36.866964,28.678586 | Türkiye | Dalyan, Muğla | 26.4.2014 | https://www.gbif.org/ |
| *Mauremys rivulata* | 36.831826,28.644672 | Türkiye | Dalyan, Muğla | 22.4.2014 | https://www.gbif.org/ |
| *Mauremys rivulata* | 36.844158,28.65972 | Türkiye | Dalyan, Muğla | 29.4.2014 | https://www.gbif.org/ |
| *Mauremys rivulata* | 36.89033,28.64382 | Türkiye | Dalyan, Muğla | 21.5.2013 | https://www.gbif.org/ |
| *Mauremys rivulata* | 36.628909,35.449173 | Türkiye | Adana | 29.9.2011 | https://www.gbif.org/ |
| *Mauremys rivulata* | 36.839257,28.636208 | Türkiye | Dalyan, Muğla | 25.6.2012 | https://www.gbif.org/ |
| *Mauremys rivulata* | 36.843911,28.676033 | Türkiye | Dalyan, Muğla | 22.10.2013 | https://www.gbif.org/ |
| *Mauremys rivulata* | 36.744119,31.486359 | Türkiye | Manavgat, Antalya | 11.5.2013 | https://www.gbif.org/ |
| *Mauremys rivulata* | 36.403299,33.866416 | Türkiye | Silifke, Mersin | 13.6.2008 | https://www.gbif.org/ |
| *Mauremys rivulata* | 36.82,28.62 | Türkiye | Dalyan, Muğla | 21.10.2013 | https://www.gbif.org/ |
| *Mauremys rivulata* | 36.671796,34.41889 | Türkiye | Mersin | 11.8.2010 | https://www.gbif.org/ |
| *Mauremys rivulata* | 36.577078,34.208266 | Türkiye | Mersin | 14.6.2008 | https://www.gbif.org/ |
| *Mauremys rivulata* | 36.744119,31.486359 | Türkiye | Manavgat, Antalya | 11.5.2013 | https://www.gbif.org/ |
| *Mauremys rivulata* | 36.535436,32.032714 | Türkiye | Alanya | 27.4.2015 | https://www.gbif.org/ |
| *Mauremys rivulata* | 36.78,28.62 | Türkiye | Dalyan, Muğla | 4.5.2014 | https://www.gbif.org/ |
| *Mauremys rivulata* | 36.900327,28.622034 | Türkiye | Dalyan, Muğla | 21.4.2014 | https://www.gbif.org/ |
| *Mauremys rivulata* | 36.535436,32.032714 | Türkiye | Alanya | 27.4.2015 | https://www.gbif.org/ |
| *Mauremys rivulata* | 37.201064,36.076337 | Türkiye | Adana | 17.6.2014 | https://www.gbif.org/ |
| *Mauremys rivulata* | 36.843362,28.668995 | Türkiye | Dalyan, Muğla | 12.10.2013 | https://www.gbif.org/ |
| *Mauremys rivulata* | 36.321367,33.903261 | Türkiye | Göksu Delta | 26.5.2013 | https://www.gbif.org/ |
| *Mauremys rivulata* | 36.82,28.61 | Türkiye | Dalyan, Muğla | 13.10.2013 | https://www.gbif.org/ |
| *Mauremys rivulata* | 36.784619,35.447597 | Türkiye | Adana | 30.5.2013 | https://www.gbif.org/ |
| *Mauremys rivulata* | 36.744119,31.486359 | Türkiye | Manavgat, Antalya | 11.5.2013 | [https://www.gbif.org/](http://www.turkherptil.org/) |
| *Mauremys rivulata* | 36.08,32.89 | Türkiye | Anamur, Mersin | 9.12.2012 | [https://www.gbif.org/](http://www.turkherptil.org/) |
| *Mauremys rivulata* | 36.843634,28.63182 | Türkiye | Dalyan, Muğla | 21.5.2013 | https://www.gbif.org/ |
| *Mauremys rivulata* | 36.744119,31.486359 | Türkiye | Manavgat, Antalya | 11.5.2013 | https://www.gbif.org/ |
| *Mauremys rivulata* | 36.84343,28.631916 | Türkiye | Dalyan, Muğla | 23.5.2013 | https://www.gbif.org/ |
| *Mauremys rivulata* | 36.840026,28.635086 | Türkiye | Dalyan, Muğla | 14.6.2012 | https://www.gbif.org/ |
| *Mauremys rivulata* | 36.949293,31.16824 | Türkiye | Aspendos, Antalya | 27.3.2012 | https://www.gbif.org/ |
| *Mauremys rivulata* | 36.323424,33.902264 | Türkiye | GÖksu Delta | 14.7.2010 | https://www.gbif.org/ |
| *Mauremys rivulata* | 37.087629,27.617435 | Türkiye | Muğla | 12.8.2011 | https://www.gbif.org/ |
| *Mauremys rivulata* | 36.666642,30.535099 | Türkiye | Antalya | 8.5.2011 | https://www.gbif.org/ |
| *Mauremys rivulata* | 36.68333,30.527636 | Türkiye | Antalya | 2.8.2020 | https://www.gbif.org/ |
| *Mauremys rivulata* | 36.848755,28.284268 | Türkiye | Muğla | 29.6.2020 | https://www.gbif.org/ |
| *Mauremys rivulata* | 36.985587,28.261265 | Türkiye | Muğla | 17.5.2009 | https://www.gbif.org/ |
| *Mauremys rivulata* | 36.989358,28.251566 | Türkiye | Muğla | 7.6.2009 | https://www.gbif.org/ |
| *Mauremys rivulata* | 36.822862,28.21142 | Türkiye | Muğla | 18.5.2020 | https://www.gbif.org/ |
| *Mauremys rivulata* | 36.32477,30.172152 | Türkiye | Antalya | 4.5.2014 | https://www.gbif.org/ |
| *Mauremys rivulata* | 36.425224,30.44669 | Türkiye | Antalya | 7.5.2015 | https://www.gbif.org/ |
| *Mauremys rivulata* | 36.764801,31.448235 | Türkiye | Antalya | 27.7.2004 | https://www.gbif.org/ |
| *Mauremys rivulata* | 36.810262,31.34236 | Türkiye | Antalya | 13.9.2019 | https://www.gbif.org/ |
| *Mauremys rivulata* | 36.810475,31.342162 | Türkiye | Antalya | 13.9.2019 | https://www.gbif.org/ |
| *Mauremys rivulata* | 36.810282,31.342338 | Türkiye | Antalya | 13.9.2019 | https://www.gbif.org/ |
| *Mauremys rivulata* | 36.80469,31.352303 | Türkiye | Antalya | 10.9.2019 | https://www.gbif.org/ |
| *Mauremys rivulata* | 36.826633,28.620718 | Türkiye | Muğla | 6.10.2008 | https://www.gbif.org/ |
| *Mauremys rivulata* | 36.986073,28.25284 | Türkiye | Muğla | 19.8.2019 | https://www.gbif.org/ |
| *Mauremys rivulata* | 38.185097,26.804125 | Türkiye | Izmir | 1.6.2019 | https://www.gbif.org/ |
| *Mauremys rivulata* | 37.949579,27.363913 | Türkiye | Izmir | 26.3.2019 | https://www.gbif.org/ |
| *Mauremys rivulata* | 36.680486,29.090779 | Türkiye | Muğla | 3.8.2014 | https://www.gbif.org/ |
| *Mauremys rivulata* | 36.349537,30.270894 | Türkiye | Antalya | 24.4.2013 | https://www.gbif.org/ |
| *Mauremys rivulata* | 36.349553,30.270778 | Türkiye | Antalya | 24.4.2013 | https://www.gbif.org/ |
| *Mauremys rivulata* | 36.707732,28.698694 | Türkiye | Muğla | 9.9.2009 | https://www.gbif.org/ |
| *Mauremys rivulata* | 36.707732,28.698694 | Türkiye | Muğla | 10.9.2009 | https://www.gbif.org/ |
| *Mauremys rivulata* | 37.451777,29.680871 | Türkiye | Burdur | 17.7.2016 | https://www.gbif.org/ |
| *Mauremys rivulata* | 36.83429,28.6446 | Türkiye | Muğla | 15.5.2013 | https://www.gbif.org/ |
| *Mauremys rivulata* | 40.345555555, 29.257777777 | Türkiye | Bursa | 7.07.2013 | Keleş, 2019 |
| *Mauremys rivulata* | 40.125898,26.289922 | Türkiye | Çanakkale | 27.03.2007 | Museum Collection |
| *Mauremys rivulata* | 40.101202,26.376480 | Türkiye | Çanakkale | 3.04.2018 | Museum Collection |
| *Mauremys rivulata* | 40.384679,26.723963 | Türkiye | Çanakkale | 30.04.2009 | Museum Collection |
| *Mauremys rivulata* | 40.604462,26.847873 | Türkiye | Çanakkale | 10.10.2009 | Museum Collection |
| *Mauremys rivulata* | 40.147542,25.928105 | Türkiye | Çanakkale | 4.07.2019 | Museum Collection |
| *Mauremys rivulata* | 37.42260,30.55399 | Türkiye | Bucak, Burdur | 2000 | Ayaz and Budak, 2008 |
| *Mauremys rivulata* | 36.06076,36.01710 | Türkiye | Samandağ, Hatay | 2002 | Ayaz and Budak, 2008 |
| *Mauremys rivulata* | 36.9541,36.5715 | Türkiye | İslahiye, Gaziantep | 2001-2002 | Ayaz and Budak, 2008 |
| *Mauremys rivulata* | 36.66562,35.56749 | Türkiye | Yumurtalık, Adana | 2001-2002 | Ayaz and Budak, 2008 |
| *Mauremys rivulata* | 36.7894,36.4186 | Türkiye | Hassa, Hatay | 2001-2002 | Ayaz and Budak, 2008 |
| *Mauremys rivulata* | 37.1992,36.5731 | Türkiye | Bahçe, Osmaniye | 2001-2002 | Ayaz and Budak, 2008 |
| *Mauremys rivulata* | 36.5482,36.2043 | Türkiye | İskenderun, Hatay | 2001-2002 | Ayaz and Budak, 2008 |
| *Mauremys rivulata* | 37.9526,30.8751 | Türkiye | Eğirdir Lake, Isparta | 2001-2002 | Ayaz and Budak, 2008 |
| *Mauremys rivulata* | 36.8240,31.5119 | Türkiye | Manavgat, Antalya | 2001-2002 | Ayaz and Budak, 2008 |
| *Mauremys rivulata* | 37.1456,31.6569 | Türkiye | Akseki, Antalya | 2001-2002 | Ayaz and Budak, 2008 |
| *Mauremys rivulata* | 40.17189,25.85828 | Türkiye | Gökçeada | 2014 | Bayrakcı et al., 2016 |
| *Mauremys rivulata* | 37.6705,27.3356 | Türkiye | Söke, Aydın | 2010-2011 | Bayrakcı et al., 2015 |
| *Mauremys rivulata* | 41.46930,32.25471 | Türkiye | Bartın | 2016 | Çakmak et al., 2017 |
| *Mauremys rivulata* | 39.82079,26.04368 | Türkiye | Bozcaada, Çanakkale | 20.02.2022 | Özgül et al., 2022 |
| *Mauremys rivulata* | 37.62563,28.06743 | Türkiye | Çine, Aydın | 2011-2012 | Özcan and Üzüm, 2014 |
| *Mauremys rivulata* | 36.700726,27.675086 | Türkiye | Datça, Muğla | 2017-2019 | Yakin et al., 2024 |
| *Mauremys caspica* | 39.368218,34.586194 | Türkiye | Kırşehir | 18.08.2016 | Field study |
| *Mauremys caspica* | 39.4463,38.6051 | Türkiye | Keban Dam, Elazığ | 2004 | Özcan and Sarıeyyüpoğlu, 2009 |
| *Mauremys caspica* | 37.1752,40.8145 | Türkiye | Nusaybin, Mardin | 2001 | Cihan et al., 2003 |
| *Mauremys caspica* | 38.39277,43.17334 | Türkiye | Van Lake, Van | 2014-2017 | Adızel et al., 2017 |
| *Mauremys caspica* | 38.3356,36.8382 | Türkiye | Kahramanmaraş | 2016-2018 | Yalçınkaya et al., 2022 |
| *Mauremys caspica* | 38.4428,37.0147 | Türkiye | Kahramanmaraş | 2016-2018 | Yalçınkaya et al., 2022 |
| *Mauremys caspica* | 38.3293,37.2732 | Türkiye | Kahramanmaraş | 2016-2018 | Yalçınkaya et al., 2022 |
| *Mauremys caspica* | 37.8975,36.8350 | Türkiye | Kahramanmaraş | 2016-2018 | Yalçınkaya et al., 2022 |
| *Mauremys caspica* | 37.7146,37.3489 | Türkiye | Kahramanmaraş | 2016-2018 | Yalçınkaya et al., 2022 |
| *Mauremys caspica* | 37.9349,34.8344 | Türkiye | Niğde | 2008 | Göçmen et al., 2008 |
| *Mauremys caspica* | 37.5850,37.6290 | Türkiye | Adıyaman | 2016-2018 | Yıldız et al. 2023 |
| *Mauremys caspica* | 37.5330,37.8434 | Türkiye | Adıyaman | 2016-2018 | Yıldız et al. 2023 |
| *Mauremys caspica* | 37.5062,38.1208 | Türkiye | Adıyaman | 2016-2018 | Yıldız et al. 2023 |
| *Mauremys caspica* | 37.6717,38.2895 | Türkiye | Adıyaman | 2016-2018 | Yıldız et al. 2023 |
| *Mauremys caspica* | 37.8214,38.6205 | Türkiye | Adıyaman | 2016-2018 | Yıldız et al. 2023 |
| *Mauremys caspica* | 37.9554,38.9341 | Türkiye | Adıyaman | 2016-2018 | Yıldız et al. 2023 |
| *Mauremys caspica* | 36.8268,37.7299 | Türkiye | Gaziantep | 2005-2008 | Özuslu and Tel, 2010 |
| *Mauremys caspica* | 39.5505,42.9929 | Türkiye | Ağrı | 2014 | Yıldız et al., 2018 |
| *Mauremys caspica* | 39.1438,42.8605 | Türkiye | Ağrı | 2014 | Yıldız et al., 2018 |
| *Mauremys caspica* | 39.5536,43.7967 | Türkiye | Ağrı | 2014 | Yıldız et al., 2018 |
| *Mauremys caspica* | 37.3060,40.8555 | Türkiye | Derik, Mardin | 16.05.2022 | http://turkherptil.org/ |
| *Mauremys caspica* | 37.8198,40.1652 | Türkiye | Hevsel Gardens, Diyarbakır | 13.02.2022 | http://turkherptil.org/ |
| *Mauremys caspica* | 37.4100,42.1575 | Türkiye | İdil, Şırnak | 5.04.2021 | http://turkherptil.org/ |
| *Mauremys caspica* | 38.5007,42.0661 | Türkiye | Güroymak, Bitlis | 1.06.2015 | http://turkherptil.org/ |
| *Mauremys caspica* | 39.7743,33.7500 | Türkiye | Kırıkkale | 20.04.2016 | http://turkherptil.org/ |
| *Mauremys caspica* | 37.4226,38.8191 | Türkiye | Şanlıurfa | 3.11.2018 | http://turkherptil.org/ |
| *Mauremys caspica* | 39.8752,44.1529 | Türkiye | Iğdır | 4.09.2017 | http://turkherptil.org/ |
| *Mauremys caspica* | 38.0373,39.9161 | Türkiye | Gözegöl Lake, Diyarbakır | 6.10.2016 | http://turkherptil.org/ |
| *Mauremys caspica* | 37.7725,38.4156 | Türkiye | İncebağ, Adıyaman | 17.04.2016 | http://turkherptil.org/ |
| *Mauremys caspica* | 38.0594,39.0665 | Türkiye | Gerger, Adıyaman | 12.06.2015 | http://turkherptil.org/ |
| *Mauremys caspica* | 37.6591,38.5401 | Türkiye | Samsat, Adıyaman | 8.04.2015 | http://turkherptil.org/ |
| *Mauremys caspica* | 39.7779,32.9450 | Türkiye | Göksu Park, Ankara | 7.08.2011 | http://turkherptil.org/ |
| *Mauremys caspica* | 38.4539,38.1212 | Türkiye | Malatya | 17.07.2014 | http://turkherptil.org/ |
| *Mauremys caspica* | 39.2829,39.1993 | Türkiye | Güneykonak, Tunceli | 28.05.2014 | http://turkherptil.org/ |
| *Mauremys caspica* | 39.7495,39.8519 | Türkiye | Çaykent, Erzincan | 4.08.2013 | http://turkherptil.org/ |
| *Mauremys caspica* | 39.0039,43.2045 | Türkiye | Erciş, Van | 19.05.2013 | http://turkherptil.org/ |
| *Mauremys caspica* | 39.7322,39.6627 | Türkiye | Ekşisu, Erzincan | 25.08.2012 | http://turkherptil.org/ |
| *Mauremys caspica* | 39.3428,39.1268 | Türkiye | Ada Village, Tunceli | 22.07.2012 | http://turkherptil.org/ |
| *Mauremys caspica* | 38.7218,41.5337 | Türkiye | Sungu, Muş | 16.06.2012 | http://turkherptil.org/ |
| *Mauremys caspica* | 38.4539,39.2797 | Türkiye | Sivrice, Elazığ | 11.05.2012 | http://turkherptil.org/ |
| *Mauremys caspica* | 37.0794,37.9415 | Türkiye | Birecik, Şanlıurfa | 2.05.2012 | http://turkherptil.org/ |
| *Mauremys caspica* | 37.5002,40.9568 | Türkiye | Savur, Mardin | 8.04.2012 | http://turkherptil.org/ |
| *Mauremys caspica* | 37.0258,37.7697 | Türkiye | Nizip, Gaziantep | 29.09.2010 | http://turkherptil.org/ |
| *Mauremys caspica* | 38.498474,43.328018 | Türkiye | Van Lake, Van | 29.05.2013 | https://www.gbif.org/ |
| *Mauremys caspica* | 38.91618,43.548817 | Türkiye | Van Lake, Van | 31.05.2011 | https://www.gbif.org/ |
| *Mauremys caspica* | 38.91618,43.548817 | Türkiye | Van Lake, Van | 24.05.2011 | https://www.gbif.org/ |
| *Mauremys caspica* | 38.91618,43.548817 | Türkiye | Van Lake, Van | 22.05.2011 | https://www.gbif.org/ |
| *Mauremys caspica* | 38.91618,43.548817 | Türkiye | Van Lake, Van | 4.06.2011 | https://www.gbif.org/ |
| *Mauremys caspica* | 38.91618,43.548817 | Türkiye | Van Lake, Van | 1.06.2011 | https://www.gbif.org/ |
| *Mauremys caspica* | 38.689796,43.577271 | Türkiye | Van | 27.05.2011 | https://www.gbif.org/ |
| *Mauremys caspica* | 38.697166,43.647457 | Türkiye | Van | 29.05.2013 | https://www.gbif.org/ |
| *Mauremys caspica* | 38.91618,43.548817 | Türkiye | Van | 3.06.2011 | https://www.gbif.org/ |
| *Mauremys caspica* | 39.004978,43.469894 | Türkiye | Van | 25.05.2011 | https://www.gbif.org/ |
| *Mauremys caspica* | 39.155491,42.32045 | Türkiye | Murat River | 26.05.2013 | https://www.gbif.org/ |
| *Mauremys caspica* | 38.10455,41.218333 | Türkiye | Batman | 31.07.2001 | https://www.gbif.org/ |
| *Mauremys caspica* | 37.159,39.035233 | Türkiye | Colap Deresi, Şanlıurfa | 8.09.2000 | https://www.gbif.org/ |
| *Mauremys caspica* | 37.159,39.035233 | Türkiye | Colap Deresi, Şanlıurfa | 8.09.2000 | https://www.gbif.org/ |
| *Mauremys caspica* | 37.159,39.035233 | Türkiye | Colap Deresi, Şanlıurfa | 8.09.2000 | https://www.gbif.org/ |
| *Mauremys caspica* | 37.786333,37.05755 | Türkiye | Kilis, Gaziantep | 9.09.2000 | https://www.gbif.org/ |
| *Mauremys caspica* | 37.786333,37.05755 | Türkiye | Kilis, Gaziantep | 9.09.2000 | https://www.gbif.org/ |
| *Mauremys caspica* | 37.786333,37.05755 | Türkiye | Kilis, Gaziantep | 9.09.2000 | https://www.gbif.org/ |
| *Mauremys caspica* | 37.786333,37.05755 | Türkiye | Kilis, Gaziantep | 9.09.2000 | https://www.gbif.org/ |
| *Mauremys caspica* | 37.786333,37.05755 | Türkiye | Kilis, Gaziantep | 9.09.2000 | https://www.gbif.org/ |
| *Mauremys caspica* | 38.985533,43.569217 | Türkiye | Ercis, Van | 5.09.2000 | https://www.gbif.org/ |
| *Mauremys caspica* | 37.912384,40.262648 | Türkiye | Diyarbakır | 10.09.2022 | https://www.gbif.org/ |
| *Mauremys caspica* | 38.697162,43.646553 | Türkiye | Van | 21.05.2022 | https://www.gbif.org/ |
| *Mauremys caspica* | 38.69715,43.64641 | Türkiye | Van | 21.05.2022 | https://www.gbif.org/ |
| *Mauremys caspica* | 38.697098,43.645766 | Türkiye | Van | 21.05.2022 | https://www.gbif.org/ |
| *Mauremys caspica* | 37.915964,40.272885 | Türkiye | Diyarbakır | 8.06.2022 | https://www.gbif.org/ |
| *Mauremys caspica* | 37.915964,40.272885 | Türkiye | Diyarbakır | 6.06.2022 | https://www.gbif.org/ |
| *Mauremys caspica* | 38.697098,43.645762 | Türkiye | Van | 21.05.2022 | https://www.gbif.org/ |
| *Mauremys caspica* | 37.915986,40.273595 | Türkiye | Diyarbakır | 11.05.2022 | https://www.gbif.org/ |
| *Mauremys caspica* | 37.914454,40.261125 | Türkiye | Diyarbakır | 22.03.2022 | https://www.gbif.org/ |
| *Mauremys caspica* | 37.914401,40.260568 | Türkiye | Diyarbakır | 22.03.2022 | https://www.gbif.org/ |
| *Mauremys caspica* | 39.053276,41.573023 | Türkiye | Mus | 22.06.2020 | https://www.gbif.org/ |
| *Mauremys caspica* | 37.895074,40.251433 | Türkiye | Diyarbakır | 13.02.2022 | https://www.gbif.org/ |
| *Mauremys caspica* | 37.893862,40.250793 | Türkiye | Diyarbakır | 13.02.2022 | https://www.gbif.org/ |
| *Mauremys caspica* | 37.917513,40.248013 | Türkiye | Diyarbakır | 12.02.2022 | https://www.gbif.org/ |
| *Mauremys caspica* | 37.914743,40.260995 | Türkiye | Diyarbakır | 7.02.2022 | https://www.gbif.org/ |
| *Mauremys caspica* | 36.998069,37.918861 | Türkiye | Gaziantep | 17.05.2011 | https://www.gbif.org/ |
| *Mauremys caspica* | 36.367115,33.977966 | Türkiye | Göksu Delta | 30.04.2011 | https://www.gbif.org/ |
| *Mauremys caspica* | 37.914667,40.268849 | Türkiye | Diyarbakır | 28.09.2021 | https://www.gbif.org/ |
| *Mauremys caspica* | 37.916001,40.273341 | Türkiye | Diyarbakır | 21.09.2021 | https://www.gbif.org/ |
| *Mauremys caspica* | 37.908051,40.249476 | Türkiye | Diyarbakır | 25.09.2021 | https://www.gbif.org/ |
| *Mauremys caspica* | 37.916074,40.273744 | Türkiye | Diyarbakır | 28.09.2021 | https://www.gbif.org/ |
| *Mauremys caspica* | 38.502295,43.338346 | Türkiye | Van | 23.07.2021 | https://www.gbif.org/ |
| *Mauremys caspica* | 37.915898,40.273323 | Türkiye | Diyarbakır | 17.08.2021 | https://www.gbif.org/ |
| *Mauremys caspica* | 37.916017,40.273372 | Türkiye | Diyarbakır | 13.08.2021 | https://www.gbif.org/ |
| *Mauremys caspica* | 37.91445,40.27032 | Türkiye | Diyarbakır | 1.08.2021 | https://www.gbif.org/ |
| *Mauremys caspica* | 39.34641,42.260184 | Türkiye | Muş | 22.06.2021 | https://www.gbif.org/ |
| *Mauremys caspica* | 37.85122,40.629057 | Türkiye | Diyarbakır | 5.06.2021 | https://www.gbif.org/ |
| *Mauremys caspica* | 37.849409,40.556755 | Türkiye | Diyarbakır | 7.06.2021 | https://www.gbif.org/ |
| *Mauremys caspica* | 37.914433,40.260577 | Türkiye | Diyarbakır | 21.04.2021 | https://www.gbif.org/ |
| *Mauremys caspica* | 37.914463,40.261492 | Türkiye | Diyarbakır | 21.04.2021 | https://www.gbif.org/ |
| *Mauremys caspica* | 39.916378,44.294629 | Türkiye | Iğdır | 9.04.2021 | https://www.gbif.org/ |
| *Mauremys caspica* | 37.337502,41.74659 | Türkiye | Şırnak | 5.04.2021 | https://www.gbif.org/ |
| *Mauremys caspica* | 39.939386,44.256374 | Türkiye | Iğdır | 6.04.2021 | https://www.gbif.org/ |
| *Mauremys caspica* | 39.916378,44.294629 | Türkiye | Iğdır | 4.04.2021 | https://www.gbif.org/ |
| *Mauremys caspica* | 37.33794,41.746397 | Türkiye | Şırnak | 5.04.2021 | https://www.gbif.org/ |
| *Mauremys caspica* | 37.914748,40.259766 | Türkiye | Diyarbakır | 28.03.2021 | https://www.gbif.org/ |
| *Mauremys caspica* | 39.908097,44.290501 | Türkiye | Iğdır | 24.03.2021 | https://www.gbif.org/ |
| *Mauremys caspica* | 37.915408,40.264818 | Türkiye | Diyarbakır | 13.03.2021 | https://www.gbif.org/ |
| *Mauremys caspica* | 37.914265,40.260435 | Türkiye | Diyarbakır | 6.03.2021 | https://www.gbif.org/ |
| *Mauremys caspica* | 37.914638,40.26061 | Türkiye | Diyarbakır | 6.03.2021 | https://www.gbif.org/ |
| *Mauremys caspica* | 39.873902,44.516055 | Türkiye | Iğdır | 26.08.2020 | https://www.gbif.org/ |
| *Mauremys caspica* | 37.914763,40.260503 | Türkiye | Diyarbakır | 11.01.2021 | https://www.gbif.org/ |
| *Mauremys caspica* | 37.914634,40.26061 | Türkiye | Diyarbakır | 11.01.2021 | https://www.gbif.org/ |
| *Mauremys caspica* | 37.914402,40.261073 | Türkiye | Diyarbakır | 21.01.2021 | https://www.gbif.org/ |
| *Mauremys caspica* | 37.915141,40.260417 | Türkiye | Diyarbakır | 15.01.2021 | https://www.gbif.org/ |
| *Mauremys caspica* | 37.914302,40.259848 | Türkiye | Diyarbakır | 8.01.2021 | https://www.gbif.org/ |
| *Mauremys caspica* | 37.914598,40.261742 | Türkiye | Diyarbakır | 7.01.2021 | https://www.gbif.org/ |
| *Mauremys caspica* | 37.006819,37.967552 | Türkiye | Sanliurfa | 4.06.2010 | https://www.gbif.org/ |
| *Mauremys caspica* | 39.074621,42.309118 | Türkiye | Muş | 5.07.2020 | https://www.gbif.org/ |
| *Mauremys caspica* | 37.914583,40.263335 | Türkiye | Diyarbakır | 27.11.2020 | https://www.gbif.org/ |
| *Mauremys caspica* | 37.914329,40.266382 | Türkiye | Diyarbakır | 27.11.2020 | https://www.gbif.org/ |
| *Mauremys caspica* | 37.915971,40.27342 | Türkiye | Diyarbakır | 24.11.2020 | https://www.gbif.org/ |
| *Mauremys caspica* | 37.918141,40.260727 | Türkiye | Diyarbakır | 24.10.2020 | https://www.gbif.org/ |
| *Mauremys caspica* | 37.918835,40.261199 | Türkiye | Diyarbakır | 24.10.2020 | https://www.gbif.org/ |
| *Mauremys caspica* | 37.91619,40.273833 | Türkiye | Diyarbakır | 7.10.2020 | https://www.gbif.org/ |
| *Mauremys caspica* | 37.915751,40.273141 | Türkiye | Diyarbakır | 29.10.2020 | https://www.gbif.org/ |
| *Mauremys caspica* | 37.917133,40.26243 | Türkiye | Diyarbakır | 22.10.2020 | https://www.gbif.org/ |
| *Mauremys caspica* | 37.916307,40.273913 | Türkiye | Diyarbakır | 10.10.2020 | https://www.gbif.org/ |
| *Mauremys caspica* | 36.981472,37.974816 | Türkiye | Birecik | 17.05.2014 | https://www.gbif.org/ |
| *Mauremys caspica* | 37.047608,37.973168 | Türkiye | Birecik | 13.05.2012 | https://www.gbif.org/ |
| *Mauremys caspica* | 37.248573,37.868242 | Türkiye | Şanlıurfa | 29.09.2011 | https://www.gbif.org/ |
| *Mauremys caspica* | 37.049748,37.981646 | Türkiye | Birecik | 12.05.2012 | https://www.gbif.org/ |
| *Mauremys caspica* | 37.00502,37.968063 | Türkiye | Birecik | 6.05.2013 | https://www.gbif.org/ |
| *Mauremys caspica* | 37.047608,37.973168 | Türkiye | Birecik | 13.05.2012 | https://www.gbif.org/ |
| *Mauremys caspica* | 36.981335,37.975062 | Türkiye | Birecik | 17.05.2014 | https://www.gbif.org/ |
| *Mauremys caspica* | 37.049748,37.981646 | Türkiye | Birecik | 12.05.2012 | https://www.gbif.org/ |
| *Mauremys caspica* | 38.752308,42.49971 | Türkiye | Van | 2.06.2007 | https://www.gbif.org/ |
| *Mauremys caspica* | 38.91618,43.548817 | Türkiye | Van | 30.05.2013 | https://www.gbif.org/ |
